# Multimodal Digital Therapeutics Enhanced by Task Design and AI for Attention-Deficit/Hyperactivity Disorder Core Symptoms and Executive Functions in Children and Adolescents: Systematic Review and Network Meta-Analysis of Randomized Controlled Trials

**DOI:** 10.2196/95043

**Published:** 2026-08-18

**Authors:** Zhixiang Hao, Hongli Xu, Chen Wang, Bingjie Wang, Zhengang Qiu

**Affiliations:** 1 College of Rehabilitation Medicine, Shandong University of Traditional Chinese Medicine, NO. 4655 Da Xue Road, Changqing District, Jinan, Shandong, 250355, China, 86 13869144597; 2 Jinan Vocational College of Nursing, Jinan, Shandong, China

**Keywords:** attention-deficit/hyperactivity disorder, digital therapeutics, AI, cognitive-motor dual-task training, network meta-analysis, PRISMA, Preferred Reporting Items for Systematic Reviews and Meta-Analyses, artificial intelligence

## Abstract

**Background:**

Attention-deficit/hyperactivity disorder (ADHD) is a prevalent neurodevelopmental disorder in children and adolescents. Digital therapeutics (DTx) show promise as nonpharmacological interventions, but the comparative efficacy of different DTx modalities remains unclear.

**Objective:**

This network meta-analysis (NMA) compared 4 DTx modalities (single-task, cognitive-motor dual-task, AI-integrated single-task, and AI-integrated cognitive-motor dual-task DTx) on core ADHD symptoms and executive functions, identified the optimal modality, and explored treatment moderators.

**Methods:**

We included randomized controlled trials (RCTs) in children and adolescents aged 4 to 17 years with ADHD diagnosed per the DSM-5 (Diagnostic and Statistical Manual of Mental Disorders [Fifth Edition]) or ICD-10 (International Classification of Diseases, Tenth Revision). We searched PubMed/MEDLINE, PsycINFO, Web of Science, EMBASE, Scopus, ProQuest Dissertations and Theses, Cochrane Library, and ClinicalTrials.gov (gray literature) to identify trials published between January 2000 to May 2026 (last search May 22, 2026) without language restrictions, supplemented by snowballing. Risk of bias was assessed with the Cochrane Risk of Bias (RoB) 2 tool. Data were synthesized using Bayesian NMA with random-effects models. The surface under the cumulative ranking curve (SUCRA) was used to rank interventions. Heterogeneity was evaluated via 95% prediction intervals (95% PI) and explored through subgroup analyses and meta-regression. Small-study effects were assessed using Egger test, and sensitivity analyses were also performed.

**Results:**

Thirty-two RCTs (2819 patients) were included. The risk of bias assessment identified a low risk in 37.5% of the studies, some concerns in 21.9% of the studies, and a high risk in 40.6% of the studies, mainly due to inadequate reporting of randomization or blinding. AI-integrated cognitive-motor dual-task DTx ranked first for all outcomes in Bayesian network meta-analysis. For the Attention-Deficit/Hyperactivity Disorder-Rating Scale (ADHD-RS; 7 studies, n=1642), SUCRA was 57.5% (mean difference [MD] –3.03, 95% credible intervals [95% CrI] –5.59 to –0.47). For the Swanson, Nolan, and Pelham Rating Scale (Version IV; SNAP-IV) inattention subscale (SNAP-IV-PI; 8 studies, n=468), SUCRA was 82.5% (MD –5.58, 95% CrI –8.76 to –2.39); for the SNAP-IV hyperactivity-impulsivity subscale (SNAP-IV-PHI; 8 studies, n=468), SUCRA was 92.6% (MD –6.84, 95% CrI –10.37 to –3.31). For the Behavior Rating Inventory of Executive Function (BRIEF; 23 studies, n=1927), SUCRA was 84.4% (MD –7.75, 95% CrI –10.06 to –5.43). In pairwise meta-analyses, the 95% PI for ADHD-RS did not cross zero (−7.19 to −0.11), whereas those for the SNAP-IV (PI subscale: −5.62 to 1.87; PHI subscale: −6.66 to 2.82) and BRIEF (−6.91 to 1.94) did, indicating limited generalizability and substantial between-study heterogeneity. Subgroup analyses suggested intervention duration as a heterogeneity source for the SNAP-IV (both subscales) and BRIEF and mean age as a heterogeneity source for the SNAP-IV-PI and BRIEF.

**Conclusions:**

This NMA provides the first dual-dimension classification framework for ADHD DTx, combining SUCRA ranking, PI, and GRADE (Grading of Recommendations Assessment, Development and Evaluation). AI-integrated cognitive-motor dual-task DTx had the highest probability of improving core symptoms and executive functions, with duration and age as potential heterogeneity sources. These findings inform clinical decision-making and DTx development, although interpretation should account for evidence limitations.

## Introduction

Attention-deficit/hyperactivity disorder (ADHD) is a neurodevelopmental disorder originating in childhood and is associated with a high prevalence rate among pediatric and adolescent populations [1]. Global epidemiological data indicate that the prevalence of ADHD is approximately 5% to 7%, making it one of the most common disorders in the field of child mental health [2]. The core clinical features of ADHD are characterized by inattention, hyperactivity, and impulsivity, frequently co-occurring with significant functional impairments in other domains, particularly executive functions [3]. In the absence of effective intervention, ADHD not only profoundly compromises academic performance, social functioning, and activities of daily living in affected individuals but its long-term consequences also substantially reduce their quality of life, ultimately impeding optimal developmental trajectories [4,5].

While conventional pharmacological and behavioral interventions have shown efficacy in treating ADHD, their clinical utility is frequently constrained by significant adverse effects, limited treatment accessibility, and suboptimal long-term patient adherence [6,7]. Digital therapeutics (DTx) have emerged as a promising nonpharmacological alternative. By embedding cognitive and behavioral tasks into interactive game-based environments, DTx can enhance engagement and motivation [8,9]. As research in this field advances, cutting-edge innovations in DTx are primarily reflected in two key dimensions.

First, in the domain of task design, intervention paradigms are evolving from traditional single-task formats (eg, early serious games) toward cognitive-motor dual-task models that more closely approximate the complexities of real-world functioning [10]. Single-task DTx typically deliver repetitive, targeted training of a single cognitive domain (eg, working memory, inhibitory control, or sustained attention) through interactive exercises. For example, Cogmed RM presents a series of visuospatial working memory tasks that gradually increase in difficulty, with the core therapeutic component being the repeated activation and strengthening of specific prefrontal cortical circuits [11]. In contrast, cognitive-motor dual-task DTx require simultaneous performance of a cognitive task and a motor task throughout the training session. A representative example is AKL-T01, which requires users to navigate a virtual environment using body movements (motor component) while responding to specific visual targets (cognitive component) [12]. The core therapeutic component of dual-task training is the coordinated activation of multiple brain regions, which more closely approximates the cognitive demands of real-world functioning.

Second, in the dimension of technological empowerment, the deep integration of AI—including machine learning algorithms, adaptive difficulty adjustment, real-time performance analytics, and personalized feedback systems—enables DTx to dynamically respond to individual differences, thereby enhancing intervention precision and therapeutic efficacy [8]. For example, the KAD-SCL-01 system uses reinforcement learning algorithms to adjust task parameters based on individual response patterns, ensuring that users remain in a state of “productive struggle” that maximizes neuroplastic changes [13].

All DTx modalities also incorporate game-based elements (eg, points, levels, rewards, and narrative contexts) as important supportive components. While game elements are not considered direct therapeutic agents, they play a critical role in enhancing user engagement, motivation, and treatment adherence, particularly in pediatric populations. A pivotal randomized controlled trial published in The Lancet by Kollins et al [12] demonstrated that an AI-integrated cognitive-motor dual-task DTx (AKL-T01) effectively improved attention and executive functions in individuals with ADHD [12]. This finding is consistent with previous conclusions, which showed that AI-driven adaptive algorithms significantly enhance treatment adherence and the magnitude of cognitive improvement in children with ADHD [14].

However, despite this potential, the existing evidence on DTx remains fragmented. Many reviews have focused on the therapeutic effects of a specific type of DTx or have been confined to a single technological dimension, failing to systematically compare the relative efficacy among different design paradigms and technological approaches within a unified framework [8,15]. More importantly, although previous traditional meta-analyses have confirmed the overall effectiveness of game-based DTx for individuals with ADHD, they have not conducted direct and indirect comparisons of multimodal DTx from the perspectives of task design (single-task vs cognitive-motor dual-task) and technological empowerment (non–AI-integrated vs AI-integrated) [16-18]. Consequently, it remains unclear which modality of DTx yields the optimal therapeutic effects for improving core symptoms and executive functions in children and adolescents with ADHD when considered from the dimensions of task design and technological empowerment. Clarifying this issue is essential for guiding clinical practice and informing future research.

This study is the first network meta-analysis (NMA) to systematically compare the 4 DTx modalities within the dual framework of task design and AI empowerment. In contrast to previous pairwise meta-analyses that only compared DTx against controls or examined a single DTx type, our approach enables simultaneous direct and indirect comparisons across all 4 modalities; identifies the optimal intervention using SUCRA ranking; and explores intervention duration, age, and sex as potential moderators of treatment outcomes. This work is particularly timely, given the rapid expansion of AI-enabled DTx and the pressing need for evidence to support clinical decision-making and guide the development of next-generation DTx.

This study is explicitly designed as an efficacy-focused systematic review and NMA to (1) compare the relative efficacy of the 4 DTx modalities on core ADHD symptoms and executive functions within this dual framework, rather than to assess the feasibility of implementation or evaluate real-world effectiveness; (2) identify the optimal intervention modality based on the surface under the cumulative ranking curve (SUCRA) values; and (3) explore the influence of factors such as intervention duration, age, and sex on treatment outcomes. These findings may contribute to the evolving evidence base for DTx in ADHD and help inform both clinical decision-making and the design of future interventions.

## Methods

The study was conducted in strict accordance with the principles and requirements of the PRISMA (Preferred Reporting Items for Systematic Reviews and Meta-Analyses) guidelines for systematic reviews and NMAs (see Checklist 1) [19].

### Information Sources and Search Strategy

We conducted and reported the literature search in accordance with the PRISMA-S (Preferred Reporting Items for Systematic Reviews and Meta-Analyses Literature Search Extension) statement [20]. A comprehensive systematic search was performed across 8 electronic databases: PubMed/MEDLINE, PsycINFO, Web of Science, EMBASE, Scopus, ProQuest Dissertations and Theses, Cochrane Library, and ClinicalTrials.gov (for gray literature). The search covered January 1, 2000, to May 22, 2026, with no language restrictions.

We used a combination of controlled vocabulary (eg, MeSH) and free-text terms, optimized for each database platform. The full, exact search strategy for each database is provided in Multimedia Appendix 1.

Database searches were supplemented by citation searching (snowballing), that is, screening the reference lists of all included studies and relevant systematic reviews. No additional searches of conference proceedings, clinical trial registries beyond ClinicalTrials.gov, or expert contacts were performed.

All retrieved records were merged and deduplicated using reference management software prior to screening.

### Selection Process

Two reviewers (HX and BW) independently screened all retrieved records at the title and abstract stage, followed by full-text screening for potentially eligible studies. Any discrepancies regarding eligibility were resolved through discussion or by consulting a third reviewer (CW) until consensus was reached. The flow of study selection was documented and reported in accordance with PRISMA 2020 guidelines.

### Eligibility Criteria

#### Inclusion Criteria

Inclusion criteria were established according to the PICOS (participants, intervention, comparator, outcome, and studies) framework as follows:

- Participants: we included randomized controlled trials (RCTs) that enrolled children and adolescents, aged 4 to 17 years, with a formal diagnosis of ADHD (any subtype) according to DSM-5 (Diagnostic and Statistical Manual of Mental Disorders [Fifth Edition]) [21] or ICD-10 (International Classification of Diseases, Tenth Revision) [22] criteria.
- Intervention: the experimental group received any modality of DTx. Modalities were classified according to a predefined framework based on “task design” (single-task vs cognitive-motor dual-task) and “AI empowerment” (yes vs no). Single-task DTx was defined as interventions focusing on a single cognitive domain at a time (eg, working memory training or attention training) without concurrent motor task requirements. Interventions that required sequential completion of separate cognitive and motor tasks were also classified as single-task DTx. In contrast, cognitive-motor dual-task DTx was predefined as requiring simultaneous performance of a cognitive task (eg, attentional control, working memory updating, and inhibitory control) and a motor task (eg, gross motor movement, fine motor coordination, and balance control) throughout the training session. AI integration was defined as the incorporation of machine learning algorithms that dynamically analyze individual user performance data in real time to personalize intervention parameters. Specifically, an intervention was classified as AI-integrated DTx if it met at least one of the following criteria: (1) it used supervised learning, reinforcement learning, or decision tree algorithms to adjust task difficulty based on individual performance trajectories; (2) it generated personalized feedback or content recommendations based on real-time analysis of user response patterns; (3) it adapted training sequences to target specific cognitive weaknesses identified through ongoing performance assessment. Simply preprogrammed difficulty adjustment, generic game-based feedback, or static branching logic was not considered sufficient for the AI-integrated DTx classification. Two independent reviewers (ZH and BW) classified all included interventions according to the above criteria. Any discrepancies were resolved through discussion with a third reviewer (CW) until consensus was reached.
- Comparator: control groups were first categorized according to whether they involved medication. Medication comparators (MED) included any treatment that contained medication, such as stimulants, nonstimulants, or usual care that was predominantly pharmacological. Nonmedication comparators (non-MED) included conditions that did not involve medication, such as waitlist, no treatment, placebo or sham stimulation, cognitive training, behavioral therapy, psychosocial therapy, conventional rehabilitation training, or usual care without medication. DTx modalities were classified according to the predefined “task design” (single-task vs cognitive-motor dual-task) and “AI empowerment” (yes vs no) framework.
- Outcomes: at least one of the following domains was reported using objective or subjective assessment measures:
  1. Core symptoms of ADHD in children or adolescents, assessed using the Attention-Deficit/Hyperactivity Disorder Rating Scale (ADHD-RS) and the Swanson, Nolan, and Pelham Rating Scale, Version IV (SNAP-IV)—for the SNAP-IV, particular attention was given to the inattention (PI) and hyperactivity-impulsivity (PHI) subscales;
  2. Executive functions in children or adolescents with ADHD, assessed using the Behavior Rating Inventory of Executive Function (BRIEF);
  3. Given that DTx are typically not delivered in school settings and because the availability of teacher-reported measures was inconsistent across the relevant studies, we included only parent-reported versions of the scales in this study to ensure consistency of rater perspective across all trials.
- Study design: published or unpublished RCTs, regardless of blinding status, were included.

#### Exclusion Criteria

Exclusion criteria were as follows: (1) studies with incomplete data or those for which full texts were unavailable, (2) non-RCT designs (eg, reviews, case reports, single-arm studies), (3) studies including participants outside the specified age range of 4 to 17 years or those without a confirmed diagnosis of ADHD, (4) studies primarily targeting children and adolescents with ADHD comorbid with other primary severe psychiatric or neurodevelopmental disorders (eg, autism spectrum disorder and schizophrenia), (5) interventions primarily consisting of nondigital conventional treatments, and (6) non-original research articles (eg, protocols, commentaries, and editorials).

### Data Collection Process

Data extraction and quality assessment of the included studies were independently conducted by 2 reviewers (ZH and BW). Any disagreements were resolved through discussion and consultation with a third reviewer (ZQ). The study selection process proceeded as follows: first, titles were screened to exclude irrelevant studies. Subsequently, abstracts and full texts were further reviewed, and studies were evaluated against the predefined inclusion and exclusion criteria to determine eligibility. When critical information was missing, the corresponding authors of the original studies were contacted via email or telephone.

The participant samples across all included studies were mutually independent, and no duplicate counting of participants occurred in the NMA. For multiarm trials, only data from arms that fulfilled the inclusion criteria were included in the analyses.

### Data Items

The following data were extracted: (1) first author and year of publication; (2) study design; (3) age of participants; (4) sample size; (5) IQ scores of participants (assessed using the Wechsler Intelligence Scale for Children, Fourth Edition [WISC-IV]); (6) sex ratio (male/female); (7) interventions in the experimental and control groups; (8) intervention duration; and (9) outcome measures. All extracted data were entered into a Microsoft Excel spreadsheet.

### Risk of Bias Assessment in Studies

We assessed the risk of bias for all eligible studies. Two reviewers (ZH and HX) independently evaluated each included RCT using the Revised Cochrane Risk of Bias (RoB 2) tool [23], and disagreements were resolved through discussion with a third reviewer (CW). RoB 2 assesses bias across 5 domains: the randomization process, deviations from intended interventions, missing outcome data, measurement of the outcome, and selection of the reported result. Each domain is judged as low risk, some concerns, or high risk according to the official RoB 2 guidance. Given that parents served as outcome assessors in all included trials and could not be blinded to treatment allocation due to the nature of the interventions, we systematically assessed this source of bias within domain 4 (measurement of the outcome) of the RoB 2 tool. The overall risk of bias was determined as follows: low risk if all domains were low risk, some concerns if at least one domain raised some concerns and no domain was high risk, and high risk if at least 1 domain was rated high risk.

### Summary Measures

All outcomes included in this study were continuous variables. The mean difference (MD) was selected as the primary effect measure. For conventional pairwise meta-analyses, the pooled effects were presented with 95% CI; for the Bayesian NMA, the estimates were quantified using posterior medians with 95% credible intervals (CrI). The MD was preferred over the standardized mean difference because all included studies used identical, widely validated assessment instruments for each outcome domain, allowing for direct comparison of effect sizes in their original clinical units and substantially enhancing the interpretability of findings for clinical practice. Core ADHD symptoms were assessed using the ADHD-RS and the SNAP-IV, including its PI and PHI subscales, while executive functions were assessed using the global executive composite score of the BRIEF parent version. For all scales, lower scores indicated less severe symptoms and better functional outcomes. For pairwise meta-analyses, statistical significance was determined by whether the 95% CI for the MD crossed zero; for Bayesian NMA, the analogous criterion was whether the 95% CrI crossed zero.

### Synthesis Methods

Data were synthesized using a Bayesian NMA with random-effects models, implemented via the R software (version 4.2.3; R Foundation for Statistical Computing) packages *gemtc* and JAGS (version 4.3.0) [24]. For conventional pairwise meta-analyses, the restricted maximum likelihood (REML) method was used to estimate the between-study heterogeneity variance, and 95% CI values were calculated using the Hartung-Knapp-Sidik-Jonkman (HKSJ) method [25,26]. Compared with the conventional DerSimonian-Laird (DL) method, this approach provides more robust statistical inference and better controls the type I error rate, which is particularly advantageous in scenarios with a limited number of studies and substantial heterogeneity [26]. All data conversions were performed in accordance with the formulas recommended in the Cochrane Handbook for Systematic Reviews of Interventions (version 6.2) [27]: $SD=\frac{\sqrt{\left(N\right)}*\left(upperlimit-lowerlimit\right)}{3.92}$ and $SD=\sqrt{N*SE}$. Specifically, CI and SE were transformed into SD. Only after all data conversions were completed were the data included in subsequent analyses. Model parameters were specified using vague prior distributions: a normal distribution with a mean of 0 and variance of 10^4^ was adopted for effect sizes (MD), while a uniform distribution with a scale parameter of 2.5 was used for the between-study heterogeneity variance. All models were implemented using 4 Markov chains, each run for 500,000 iterations, with the first 20,000 iterations discarded as the burn-in period. Thinning was applied at an interval of every 10 iterations [28]. Posterior estimates of treatment effects were summarized as posterior medians with 95% CrI. In contrast to frequentist CI, CrI provide a probabilistic interpretation of the parameter uncertainty, indicating the range within which the true effect size lies with 95% posterior probability [29]. Convergence was comprehensively assessed using the potential scale reduction factor (PSRF<1.05), alongside visual inspection of trace plots and density plots [30].

Given the potential heterogeneity among the included studies, a random-effects model was used for the NMA of both direct and indirect comparisons among interventions. This model offers a distinct advantage in that it provides relatively conservative estimates while effectively accounting for part of the heterogeneity across studies, thereby yielding more robust evidence for the conclusions [31]. Model parameters were specified using vague prior distributions to obtain posterior probability distributions for the relative effects of each treatment, thereby quantifying the uncertainty surrounding parameter estimates.

Heterogeneity was quantified using the *I*^2^ statistic and between-study variance (τ²). However, because *I*^2^ does not reflect the actual dispersion of true treatment effects across populations, we computed 95% prediction intervals (PI) based on the network estimates to quantify the real-world implications of heterogeneity [32]. In contrast to CI, which quantify the precision of the pooled average estimate, PI estimate the range of true effects that could be expected in a future individual study or clinical setting [32]. For NMA, PI were constructed using the *t*-distribution approximation method recommended by Noma et al [33].

To comprehensively explore potential sources of heterogeneity, a 3-stage strategy was implemented. First, the clinical magnitude of heterogeneity was assessed primarily via the width and coverage of PI. PI were consistently wider than their corresponding CI, indicating that between-study heterogeneity introduces additional uncertainty when extrapolating pooled effect estimates to future independent studies. The *I*^2^ statistic was reported solely as a descriptive supplementary metric. Second, subgroup analyses were conducted for categorical variables (eg, age group and sex ratio), and meta-regression analyses were performed for continuous variables (eg, intervention duration and year of publication). Third, a leave-one-out sensitivity analysis was conducted to identify individual studies exerting a dominant influence on the pooled effect sizes and to evaluate the robustness of the findings.

Network relationships among the included interventions were illustrated using a network graph. In this graph, nodes represent various interventions, with the size of each node reflecting the sample size associated with that specific intervention. Lines connecting the nodes indicate direct comparisons between pairs of interventions, and the thickness of these lines corresponds to the number of studies supporting each comparison; thicker lines represent a greater number of relevant studies [34]. A consistency model was applied when no closed loops were formed among the studies. In the presence of closed loops within the network graph, both global and local inconsistency tests were conducted [35]. Global inconsistency was evaluated by comparing the deviance information criterion (DIC) values between the consistency model and an inconsistency model (unrelated mean effects model). A smaller DIC value indicated a better model fit, and a difference of 5 or more was considered indicative of meaningful inconsistency. Local inconsistency was assessed using the node-splitting method for each closed loop in the network. For node-splitting results, a *P* value greater than .05 was considered indicative of no significant inconsistency between direct and indirect effect estimates. The consistency model was used as the primary analysis model unless significant inconsistency was identified. In the network model of this study, conventional interventions were categorized into 2 distinct nodes based on their nature: conventional nonpharmacological therapy and conventional pharmacotherapy. This separation was based on the following considerations: (1) the 2 types of interventions have fundamentally different mechanisms of action and effect sizes; (2) previous NMAs have demonstrated significant heterogeneity in their efficacy on core ADHD symptoms [36]; and (3) transitivity tests confirmed that the 2 intervention types were comparable in terms of study design and patient characteristics, satisfying the underlying assumptions of NMA.

Additionally, treatment effects were ranked by generating bar charts of SUCRA values. A larger area under the curve indicated a higher ranking of the intervention’s treatment effect [37]. Furthermore, forest plots were generated to present the results of meta-analyses.

### Reporting Bias Assessment

Small-study effects were assessed using funnel plots and Egger linear regression test for all outcome domains with 10 or more included studies, as recommended by the Cochrane Handbook for Systematic Reviews of Interventions [27]. Funnel plots and Egger test were selected due to their acceptable statistical power for detecting small-study effects in meta-analyses with a moderate number of studies [38,39]. A *P* value greater than .05 was considered indicative of no significant small-study effects. For outcome domains with fewer than 10 included studies, quantitative assessment of small-study effects was not performed due to limited statistical power. Potential biases are discussed qualitatively in the *Limitations* section.

### Certainty Assessment

Two reviewers (ZH and CW) independently assessed the certainty of evidence for each pairwise comparison, with any disagreements resolved through discussion with a third reviewer (ZQ). The certainty of evidence was evaluated using the GRADE (Grading of Recommendations Assessment, Development and Evaluation) approach adapted for NMA [40]. Evidence certainty was rated across 5 domains (risk of bias, inconsistency, indirectness, imprecision, and publication bias) as high, moderate, low, or very low in accordance with standard GRADE guidance [41].

### Protocol and Registration

This systematic review and NMA were conducted according to the protocol prospectively registered in the International Prospective Register of Systematic Reviews (PROSPERO) under registration number CRD420261304236.

### Deviations From the Registered Protocol

All deviations from the registered protocol are reported below. None materially altered the core research question, study design, or primary conclusions.

1. GRADE assessment: GRADE assessment was added post hoc to evaluate the certainty of evidence for all primary outcomes, following current best practice [42]. Complete GRADE profiles are provided in Table 1.
2. Adjustment of participant age eligibility range: the registered protocol specified an inclusion age range of 6 to 17 years. However, following the initial literature search, we revised the age range to 4 to 17 years (inclusive). This modification was made for two key reasons: (1) the DSM-5 [21] and ICD-10 both recognize a formal diagnosis of ADHD in children as young as 4 years; and (2) several high-quality RCTs meeting all other eligibility criteria had enrolled children aged 4 to 5 years. Excluding these studies would have substantially reduced the available evidence base and limited the generalizability of our findings to younger pediatric populations.
3. Search end date update: to ensure the timeliness and comprehensiveness of this review, we reran the identical search strategy across all 8 databases on May 22, 2026, and updated the search end date accordingly.
4. Statistical analysis method: we used REML for estimating between-study heterogeneity variance and HKSJ for calculating 95% CI, instead of the default DL method, to improve robustness in settings with limited studies or substantial heterogeneity.
5. PI calculation: we calculated PI where the number of studies was sufficient to quantify the real-world clinical implications of heterogeneity and to guide interpretation of pooled effect estimates.
6. Sensitivity analysis: the risk-of-bias sensitivity analysis was added post hoc to evaluate the robustness of our findings.

No other deviations from the registered protocol occurred.

**Table 1.** GRADE (Grading of Recommendations Assessment, Development and Evaluation) evidence profile for primary outcomes.

Primary outcome	Certainty assessment							Number of patients		Effect	Certainty	Importance
	Number of studies	Study design	Risk of bias	Inconsistency	Indirectness	Imprecision	Other considerations	Digital therapeutics	Conventional treatment	Absolute (95% CI)		
ADHD-RS^a^	12	Randomized clinical trials	Serious	Not serious	Not serious	Serious	One	1049	593	MD 10.1‐0.30 fewer (14.93 fewer to 4.21 more)	⨁⨁◯◯ Low	Critical
SNAP-IV-PI^b^	7	Randomized clinical trials	Serious	Not serious	Not serious	Serious	None	201	267	MD 5.27 0.04 fewer (6.84 fewer to 2.16 more)	⨁⨁◯◯ Low	Critical
SNAP-IV-PHI^c^	7	Randomized clinical trials	Serious	Not serious	Not serious	Serious	None	201	267	MD 6.67 1.92 fewer (8.55 fewer to 4.45 more)	⨁⨁◯◯ Low	Critical
BRIEF^d^	20	Randomized trials	Not serious	Not serious	Not serious	Serious	None	1188	739	MD 11.52 2.93 fewer (21.25 fewer to 9.74 more)	⨁⨁⨁◯ Moderate	Critical

a ADHD-RS: Attention-Deficit/Hyperactivity Disorder Rating Scale.

b SNAP-IV-PI: Swanson, Nolan, and Pelham Rating Scale (Version IV) inattention subscale.

c SNAP-IV-PHI: Swanson, Nolan, and Pelham Rating Scale (Version IV) hyperactivity-impulsivity subscale.

d BRIEF: Behavior Rating Inventory of Executive Function.

## Results

### Study Selection

**Figure 1.**
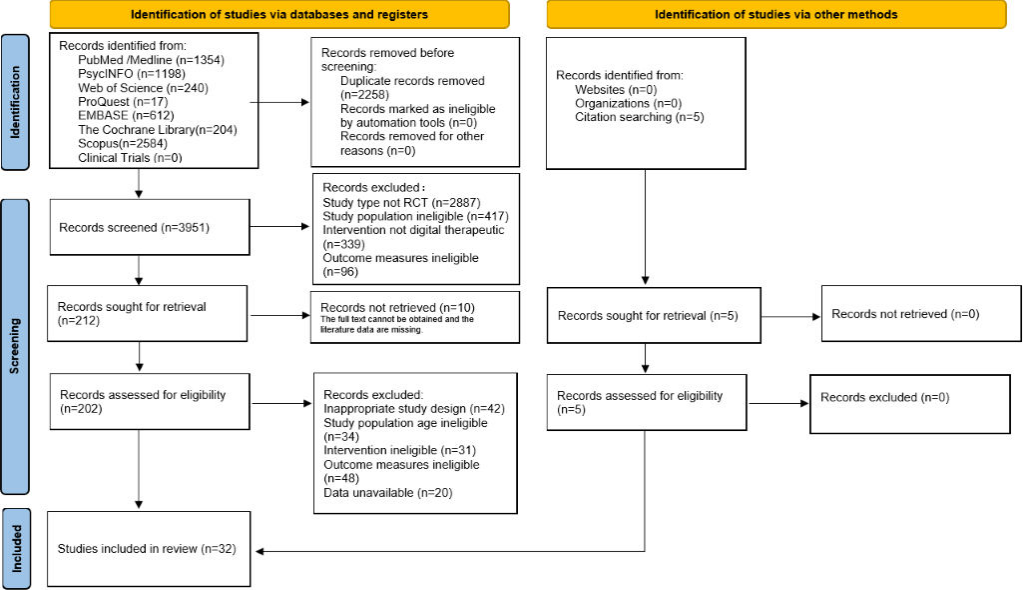
Study selection flowchart based on the PRISMA (Preferred Reporting Items for Systematic Reviews and Meta-Analyses) 2020 guidelines (flowchart adapted from Page et al [19], which is published under Creative Commons Attribution 4.0 International License [43]).

The initial database search yielded 6209 records. After removing 2258 duplicate records, the remaining 3951 records were screened based on titles and abstracts, resulting in the exclusion of 3739 articles that did not meet the inclusion criteria. An additional 10 articles were excluded because full texts could not be retrieved, leaving 202 articles for full-text assessment. During full-text evaluation, 42 articles were excluded due to ineligible study design, 34 articles were excluded due to ineligible study population age, 31 articles were excluded due to ineligible interventions, 48 articles were excluded due to outcome measures not meeting the inclusion criteria, and 20 articles were excluded due to unavailable data. Furthermore, an additional 5 articles were identified through snowballing and citation searching and included. Ultimately, a total of 32 articles were included in this NMA. The detailed screening process is presented in Figure 1.

### Study Characteristics

Table 2 shows that a total of 32 studies comprising 2819 patients were included in this NMA, with 1431 patients in the experimental groups and 1388 in the control groups. Regarding study design, 9 studies were 3-arm trials [44-52], and 23 were 2-arm trials [10,12-14,53-71]. For the 3-arm trials, only arms meeting the inclusion criteria were included in the analysis; arms that did not meet the inclusion criteria were excluded from statistical synthesis. With respect to the classification of experimental interventions, 17 studies used single-task DTx (n=559) [44-49,53-63], 4 studies used cognitive-motor dual-task DTx (n=149) [50,51,64,66], 4 studies used AI-integrated single-task DTx (n=69) [13,14,65,67], and 7 studies used AI-integrated cognitive-motor dual-task DTx (n=610) [10,12,52,68-71]. All key data required for the analyses (including sample sizes, effect estimates, and measures of variance) were fully extractable from the published articles or the available supplementary materials. Consequently, the prespecified contact procedure for missing information was not triggered, and no corresponding authors were contacted for additional data. The specific details are shown in Table 2.

**Table 2.** Characteristics of included randomized controlled trials (RCTs).

Study	Participant age (years)		Sample (treatment group/control group)	IQ	Sex ratio	Intervention		Intervention time	Outcome
	Range	Mean (SD)^a^				Treatment group	Control group		
Bikic et al (2017) [53]	14‐17	15.60 (0.99)	9/8/26	≥80	Female: 23.5%; male: 76.5%	Single-task game-based DTx:^b^ SBT^c^	CT:^d^ active placebo	Treat once a day for 30 minutes each time, 5 days a week, for a total of 7 weeks	CANTAB^e^ ADHD-RS^f^ APQ^g^
Azami et al (2016) [44]	7‐12	9.50	11/10/10	N/A^h^	Female: 0%; male: 100%	Single-task game-based DTx: CACR^i^	CT: placebo	2 months	SNAP-IV^k^ CPT^l^ TOL^m^ Digit span test Span board
							CT: MED^j^		
Bikic et al (2018) [54]	6‐13	9.95 (1.70)	35/35	≥80	Female: 17%; male: 83%	Single-task game-based DTx: CACR	CT: TAU^n^	8 weeks	AST^o^ IED^p^ SST^q^ SWM^r^ BRIEF^s^
Steiner et al (2011) [45]	11‐14	12.40 (0.90)	13/15	N/A	Female: 47.8%; male: 52.2%	Single-task game-based DTx: ACTIVATE	CT: WLC^t^	Treat twice a week for a total of 4 months	CRS-R^u^ BRIEF BASC-2^v^ IVA-CPT^w^
Rodrigo-Yanguas et al (2023) [46]	12‐22	14.38 (2.26)	35/34/35	N/A	Female: 28.1%; male: 71.9%	Single-task game-based DTx: TSTM^x^	CT: TAU	One treatment per week, for a total of 3 months	BRIEF ATENTO questionnaire BarOn Emotional Quotient Inventory: Youth Version (EQ-i:YV) SNAP-IV CPRS^y^ CPT-3^z^
						CT: active placebo			
Dovis et al (2015) [47]	8‐12	10.50 (1.30)	31/30	≥80	Female: 80.6%; male: 19.4%	Single-task game-based DTx: Braingame Brain	CT: placebo	Treatment is given 5 times a week for a total of 5 weeks	TMT^aa^ CBTT^ab^ SCWT^ac^ BRIEF Stop task DBDRS^ad^; Digit span RCPM^ae^ SPSRQ-C^af^ PedsQL^ag^ HSQ^ah^
Dang et al (2025) [55]	6‐16	9.40 (1.50)	60/64	≥80	Female: 10.5%; male: 89.5%	Single-task game-based DTx: TCT	CT: MED	8 weeks	ADHD-RS BRIEF WFIRS^ai^
Steiner et al (2014) [48]	7‐11	8.9 (1.0)	34/36	≥80	Female: 35.3%; male: 64.7%	Single-task game-based DTx: CACR	CT: TAU	Three treatments per week for a total of 5 months	Conners 3-P^aj^ BRIEF BOSS^ak^
van der Oord et al (2014) [56]	8‐12;	9.79 (1.04)	18/22	≥80	Female: 12%; male: 88%	Single-task game-based DTx: Braingame Brain	CT: MED	6 weeks	BRIEF DBDRS
Beck et al (2010) [57]	7‐17	11.75	27/24	N/A	Female: 69.2; male: 30.8%	Single-task game-based DTx: Cogmed RM	CT: WLC	5‐6 weeks	BRIEF CPRS
Bul et al (2018) [58]	8‐12	9.90 (1.26)	64/79	≥80	Female: 18%; male: 82%	Single-task game-based DTx: serious game	CT: TAU	10 weeks	BRIEF SSRS^al^ Time management skills
Bul et al (2016) [59]	8‐12	9.85 (1.26)	88/82	≥80	Female: 19.4%; male: 80.6%	Single-task game-based DTx: serious game	CT: TAU	10 weeks	BRIEF SSRS Time management skills
Meyer et al (2020) [60]	8‐11	10.33 (0.93)	20/20	N/A	Female: 30%; male: 70%	Single-task game-based DTx: computer game	CT: sham	Train 5 times a week for a total of 4 weeks of treatment	SST SNAP-IV CPRS CTRS^am^
de Oliveira Rosa et al (2021) [61]	6‐13	10.66 (1.79)	29/24	≥80	Female: 40%; male: 60%	Single-task game-based DTx: ACTIVATE	CT: sham	Treatment is given 4 times a week, 30 minutes each time, for a total of 12 weeks	SNAP-IV CGI^an^ CGAS^ao^
Jones et al (2020) [62]	7‐14	10.14 (2.02)	41/39	N/A	Female: 31.25%; male: 68.75%	Single-task game-based DTx: n-back	CT: cognitive training	Treatment is given 7 days a week for a total of 5 weeks	BRIEF CPT
Bioulac et al (2020) [49]	7‐11	8.90 (1.20)	16/16/19	≥85	Female: 19.6%; male: 80.4%	Single-task game-based DTx: VR^ap^	CT: Exergame	6‐8 weeks	ADHD-RS CPT
							CT: MED		
Kirk et al (2024) [63]	5‐9	7.60 (0.94)	28/27	≥80	Female: 30.9%; male: 69.1%	Single-task game-based DTx: Tali Train	CT: SplashLearn	Treatment is given 5 times a week, 20 minutes each time, for a total of 5 weeks	BRIEF TOVA^aq^ TEA-Ch2^ar^ C-ANT^as^ SWAN^at^ IRS^au^ CBTT Backward digit span task
Zheng et al (2025) [50]	4‐6	4.93 (0.71)	14/15/21	N/A	Female: 28%; male: 72%	Cognitive-motor dual-task DTx: double n-back	CT: social-emotional training	5 weeks	BRIEF Digit span SNAP-IV Color word span RAST-K^av^ KeyMath-3^aw^
							CT: WLC		
Wong et al (2024) [51]	6‐12	8.63 (1.90)	30/30/30	≥85	Female: 23%; male: 77%	Cognitive-motor dual-task DTx: VR	CT: cognitive training	3 weeks	BRIEF SSIS-RS^ax^
							CT: WLC		
Hilton et al (2020) [64]	8‐12	9.83 (1.48)	20/20	≥80	Female: 45%; male: 55%	Cognitive-motor dual-task DTx: PONS^ay^ + n-back	Single-task game-based DTx: n-back	Treat only once	PONS BRIEF CBCL^az^
Lim et al (2019) [66]	6‐12	8.60 (1.51)	85/87	N/A	Female: 14.5%; male: 85.5%	Cognitive-motor dual-task DTx: Cogoland	CT: WLC	20 weeks	ADHD-RS IRS CGI-I^ba^ CGAS PAERS^bb^ CBCL
Kim et al (2022) [65]	6‐13	9.27 (1.62)	15/15	N/A	Female: 20%; male: 80%	AI-integrated single-task DTx: NeuroWorld	CT: MED	4 weeks	ADHD-RS CAT^bc^ CGI CBCL
Bilan et al (2025) [14]	8‐11	9.41 (1.22)	20/21	N/A	Female: 20%; male: 80%	AI-integrated single-task DTx: KAD-SCL-01	Single-task game-based DTx: Exergame	Treat 3 times a week, 15 minutes each time, for a total of 12 weeks	BRIEF CPT-3 NEPSY II^bd^ EDAH^be^ WISC-IV^bf^
Medina et al (2021) [13]	8‐11	9.71 (1.33)	15/14	N/A	Female: 44.8%; male: 41.4%	AI-integrated single-task DTx: KAD-SCL-01	Single-task game-based DTx: Exergame	Treat 3 times a week, 15‐20 minutes each time, for a total of 12 weeks	CPT-3 BRIEF EDAH CBTT
Xu et al (2025) [67]	7‐10	8.12	19/22	≥70	Female: 17.07%; male: 82.92%	AI-integrated single-task DTx: Midjourney Vision 5	CT: traditional painting therapy	Once a week, 20‐30 minutes each time, for a total of 24 weeks	SNAP-IV WFIRS
Zhao et al (2024) [10]	6‐12	8.40 (1.30)	44/46	≥80	Female: 21.2%; male: 78.8%	AI-integrated cognitive-motor dual-task DTx: BrainFit	CT: WLC	4 weeks	SNAP-IV BRIEF
Kollins et al (2020) [12]	8‐12	9.70 (1.30)	180/168	≥80	Female: 31%; male: 69%	AI-integrated cognitive-motor dual-task DTx: AKL-T01	Single-task game-based DTx: digital word game	Treatment is given 5 days a week, 25 minutes each time, for a total of 4 weeks	TOVA API ADHD-RS IRS CGI-I BRIEF
Kollins et al (2021) [68]	8‐14	10.60 (1.77)	130/76	≥80	Female: 24.6%; male: 75.4%	AI-integrated cognitive-motor dual-task DTx: AKL-T01	CT: sham	4 weeks	ADHD-RS IRS CGI-I
Mikami et al (2025) [69]	6‐17	9.80 (2.70)	109/55	≥80	Female: 25.7%; male: 74.3%	AI-integrated cognitive-motor dual-task DTx: SDT-001	CT: TAU	6 weeks	ADHD-RS IRS BRIEF Conners 3-P
McDermot et al (2020) [70]	8‐12	9.57 (1.34)	21/19	≥70	Female: 30%; male: 70%	AI-integrated cognitive-motor dual-task DTx: FFM^bg^	CT: cognitive training	8 weeks	ADHD-RS CGI PERMP^bh^ WJ-III^bi^
Qian et al (2018) [71]	6‐12	9.00 (1.50)	18/11	≥70	Female: 0%; male: 100%	AI-integrated cognitive-motor dual-task DTx: 3D Exergame	CT: N/A	8 weeks	ADHD-RS CBCL
Mikami et al (2025) [52]	6‐17	9.60 (2.10)	108/107/46	N/A	Female: 26.9%; male: 73.1%	AI-integrated cognitive-motor dual-task DTx: SDT-001	CT: psychosocial therapy	6 weeks	ADHD-RS ACS^bj^ IRS CGI-I Conners 3-P PGA^bk^ TEAE^bl^ C-SSRS^bm^
						Single-task game-based DTx: Exergame			

a SD values are presented where available.

b DTx: digital therapeutics.

c SBT: Scientific Brain Training.

d CT: conventional treatments.

e CANTAB: Cambridge Neuropsychological Test Automated Battery.

f ADHD-RS: Attention-Deficit/Hyperactivity Disorder-Rating Scale.

g APQ: Activity Perception Questionnaire.

h N/A: not applicable.

i CACR: computer-assisted cognitive Rehabilitation.

j MED: medication.

k SNAP-IV: Swanson, Nolan, and Pelham Rating Scale, Version IV.

l CPT: continuous performance test.

m TOL: Tower of London.

n TAU: treatment as usual.

o AST: attention switching task.

p IED: intra-extra dimensional set shift.

q SST: stop-signal task.

r SWM: spatial working memory.

s BRIEF: Behavior Rating Inventory of Executive Function.

t WLC: waitlist control.

u CRS-R: Conners Rating Scales-Revised.

v BASC-2: Behavior Assessment Scales for Children-2.

w IVA-CPT: Integrated Visual and Auditory Continuous Performance Test.

x TSTM: The Secret Trail of Moon.

y CPRS: Conners Parent Rating Scale.

z CPT-3: Conners Continuous Performance Test 3.

aa TMT: Trail Making Test.

ab CBTT: Corsi Block Tapping Task.

ac SCWT: Stroop color-word test.

ad DBDRS: Disruptive Behavior Disorder Rating Scale.

ae RCPM: Raven's Coloured Progressive Matrices.

af SPSRQ-C: Sensitivity to Punishment and Sensitivity to Reward Questionnaire for Children.

ag PedsQL: Pediatric Quality of Life Inventory.

ah HSQ: Home Situations Questionnaire.

ai WFIRS: Weiss Functional Impairment Rating Scale.

aj Conners 3-P: Conners 3-Parent Assessment Report.

ak BOSS: Behavioral Observation of Students in Schools.

al SSRS: cooperation skills.

am CTRS: Conners Teacher Rating Scale.

an CGI: Clinical Global Impression.

ao CGAS: Children's Global Assessment Scale.

ap VR: virtual reality.

aq TOVA API: Test of Variables of Attention-Attention Performance Index.

ar TEA-Ch2: Test of Everyday Attention for Children-Second Edition.

as C-ANT: Child Attention Network Task.

at SWAN: Strengths and Weaknesses of ADHD Symptoms and Normal Behavior Scale.

au IRS: Impairment Rating Scale.

av RAST-K: Reading Ability Screening Test for Preschool Children.

aw KeyMath-3: KeyMath-3 Diagnostic Assessment.

ax SSIS-RS: Social Skills Improvement System-Rating Scales.

ay PONS: Profile of Nonverbal Sensitivity.

az CBCL: Child Behavior Checklist.

ba CGI-I: Clinical Global Impressions-Improvement Scale.

bb PAERS: Pediatric Adverse Event Rating Scale.

bc CAT: Comprehension Attention Test.

bd NEPSY II: Developmental Neuropsychological Assessment, Second Edition.

be EDAH: Escalas Para La Evaluación Del Trastorno Por Déficit de Atención Con Hiperactividad.

bf WISC-IV: Wechsler Intelligence Scale for Children, Fourth Edition.

bg FFM: feed-forward modeling.

bh PERMP: Permanent Product Measure of Performance.

bi WJ-III: Woodcock-Johnson Third Edition.

bj ACS: Attention Comparison Score.

bk PGA: Physician's Global Assessment.

bl TEAE: treatment-emergent adverse event.

bm C-SSRS: Columbia-Suicide Severity Rating Scale.

### Risk of Bias in Studies

**Figure 2.**
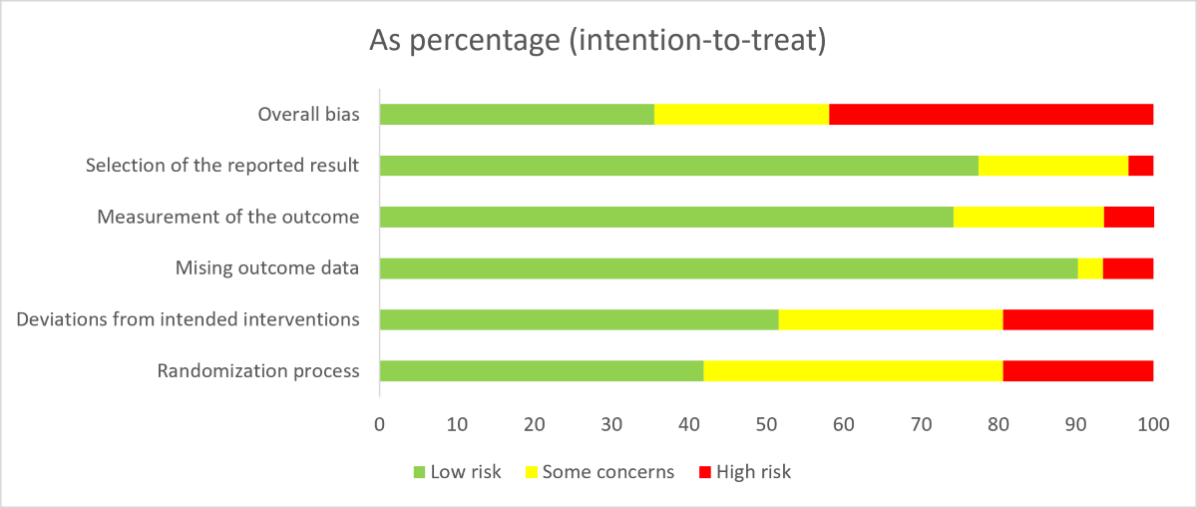
Summary of the literature quality assessment performed using the RoB 2 tool.

**Figure 3.**
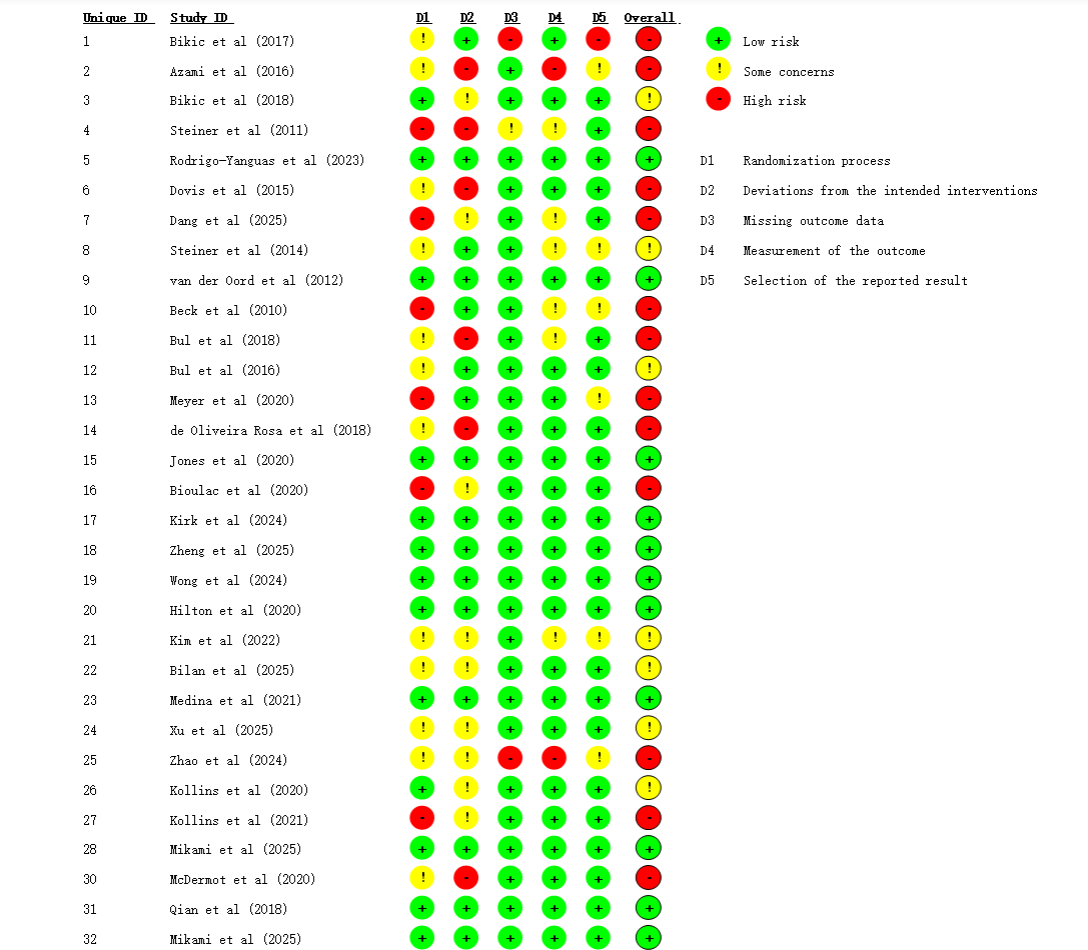
Diagram depicting the literature quality assessment performed using the RoB 2 tool [10,12-14,44-71].

The risk of bias of the included studies was assessed using the RoB 2 tool. The overall risk of bias assessment revealed that 37.5% of the studies were rated as “low risk,” 21.9% as having “some concerns,” and 40.6% as “high risk,” largely due to inadequate reporting of randomization methods and blinding procedures. A major contributing factor is that, in many studies, parents (the outcome assessors) were unable to be blinded to group allocation. Although this limitation was systematically assessed in domain 4 of RoB 2, we did not exclude these studies from the primary analysis; instead, their potential influence was evaluated post hoc through sensitivity analyses. Detailed information is presented in Figures 2 and 3 and Multimedia Appendix 2.

### Results of Syntheses

#### Evidence Network

**Figure 4.**
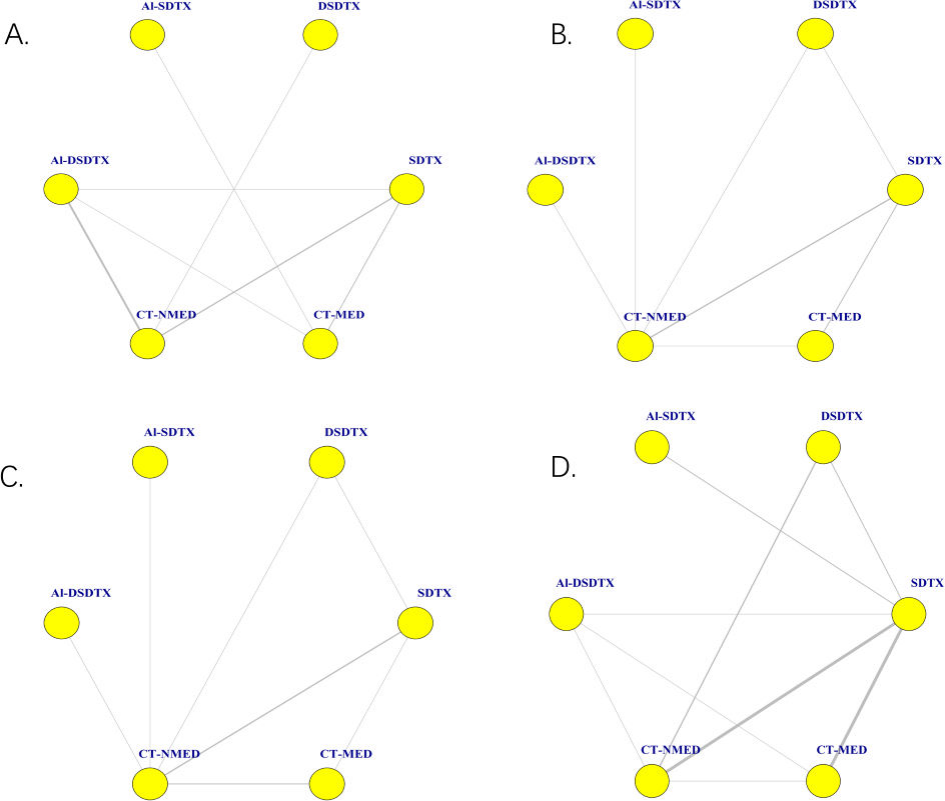
Network relationship diagrams for various primary outcome indicators: (A) ADHD-RS, (B) SNAP-IV-PI subscale, (C) SNAP-IV-PHI subscale, and (D) BRIEF. ADHD-RS: Attention-Deficit/Hyperactivity Disorder Rating Scale; AI-DSDTx: AI-integrated cognitive-motor dual-task digital therapeutics; AI-SDTx: AI-integrated single-task digital therapeutics; BRIEF: Behavior Rating Inventory of Executive Function; CT-MED: conventional treatments (MED); CT-NMED: conventional treatments (non-MED); DSDTx: cognitive-motor dual-task digital therapeutics; SDTx: single-task digital therapeutics; SNAP-IV-PHI: Swanson, Nolan, and Pelham Rating Scale (Version IV) hyperactivity-impulsivity subscale; SNAP-IV-PI: Swanson, Nolan, and Pelham Rating Scale (Version IV) inattention subscale.

To uphold the transitivity assumption, we structured the network to ensure comparability. Conventional treatments were separated into 2 nodes (non-MED and MED) based on their distinct mechanisms of action and key clinical characteristics, avoiding the risk of lumping heterogeneous controls [72]. DTx were classified by task design and AI integration to reflect shared core mechanisms within each category. Our approach is informed by high-quality NMAs of pharmacological and nonpharmacological interventions for psychiatric disorders and by recent NMAs on similar topics [73,74]. Through a series of network plots, we systematically compared the effects of the 4 DTx modalities—single-task, cognitive-motor dual-task, AI-integrated single-task, and AI-integrated cognitive-motor dual-task DTx—alongside the 2 conventional treatment nodes (non-MED and MED) as comparators, on core symptom improvement and executive functions in children and adolescents with ADHD. Figure 4A presents network plots illustrating the effects of various DTx modalities on ADHD-RS score improvement; Figure 4B-C present network plots for SNAP-IV-PI and SNAP-IV-PHI subscale score improvements, respectively; and Figure 4D presents comparative efficacy results for executive functions based on the BRIEF. Collectively, these figures provide a comprehensive comparative analysis across the 4 DTx modalities and the 2 conventional treatment nodes.

#### Inconsistency Assessment

Network plots constructed for all outcome measures in this study formed closed loops, necessitating both global and local inconsistency tests. The results of global inconsistency testing (based on DIC comparison) and local inconsistency testing (using the node-splitting method) indicated no statistically significant inconsistency across any of the comparisons (ΔDIC<5, all *P*>.05; exact *P* values are provided in Figures S1A-S1D in Multimedia Appendix 3). To further verify local consistency, we also performed a frequentist node-splitting analysis; the corresponding forest plots for direct and indirect comparisons are provided in Multimedia Appendix 3 (Figures S2A-S2D). Consistent with the Bayesian assessments, this frequentist node-splitting analysis revealed no statistically significant inconsistency between direct and indirect evidence for any comparison (all *P*>.05; exact *P* values are provided in Figures S2A-S2D in Multimedia Appendix 3), further supporting the coherence of the network evidence structure.

#### Convergence Analysis

Convergence diagnostic plots indicated that the PSRF values for all outcome measures were below 1.05. Examination of trace plots and density plots demonstrated stable fluctuations of the chains. These findings collectively indicate that the models for all outcome measures exhibited good goodness of fit and satisfactory stability. Detailed results are presented in Multimedia Appendix 3 (Figures S3A-S6B).

#### Results of Pairwise Meta-Analyses

We systematically evaluated improvements in core ADHD symptoms and executive functions using the ADHD-RS, the SNAP-IV (including both PI and PHI subscales), and the BRIEF, with lower scores indicating better treatment outcomes. Forest plots from conventional pairwise meta-analyses were generated to present the overall effect estimates with 95% CI and 95% PI.

For the ADHD-RS, the overall effect significantly favored the DTx interventions over control conditions (MD −3.65, 95% CI −4.89 to −2.41). The corresponding 95% PI (−7.19 to −0.11) remained entirely below zero, indicating that the beneficial effects of DTx on core ADHD symptoms are not only statistically significant on average but also robust and generalizable across diverse real-world populations.

For the SNAP-IV-PI subscale, the overall effect was statistically significant (MD −1.87, 95% CI −3.07 to −0.68). Similarly, for the SNAP-IV-PHI subscale, the overall effect also reached statistical significance (MD −1.92, 95% CI −3.54 to −0.31). However, the 95% PI for both subscales crossed zero (PI subscale: −5.62 to 1.87; PHI subscale: −6.66 to 2.82), suggesting that although DTx demonstrated a significant average benefit on SNAP-IV scores, the substantial between-study heterogeneity introduces considerable uncertainty when applying these interventions to individual patients in real-world clinical settings, and thus the effects could be negligible or even reverse.

For the BRIEF, the overall effect significantly favored the DTx interventions (MD −2.49, 95% CI −3.53 to −1.44). Nevertheless, the 95% PI crossed zero (−6.91 to 1.94), indicating that the average improvement in executive functions may not be reliably replicated in future heterogeneous patient populations.

**Figure 5.**
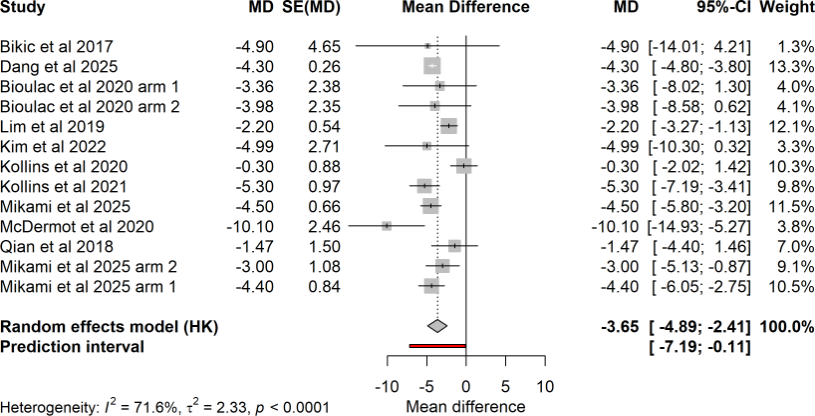
Forest plot of the ADHD-RS (Attention-Deficit/Hyperactivity Disorder Rating Scale) score [11,44,47,48,50,60,61,64-67]. MD: mean difference.

**Figure 6.**
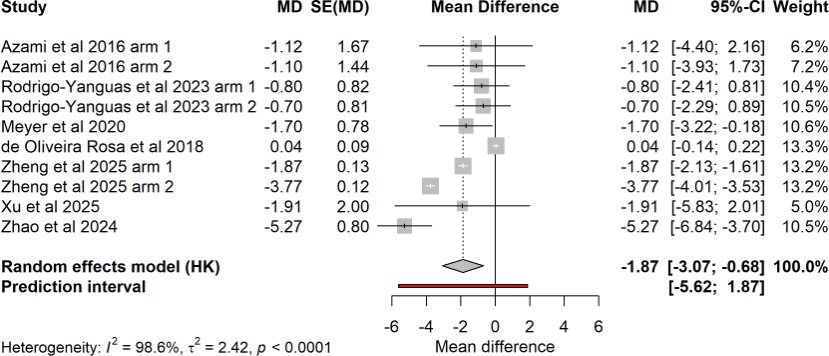
Forest plot of the SNAP-IV-PI (Swanson, Nolan, and Pelham Rating Scale [Version IV] inattention) subscale score [10,38,40,45,55,56,63]. MD: mean difference.

**Figure 7.**
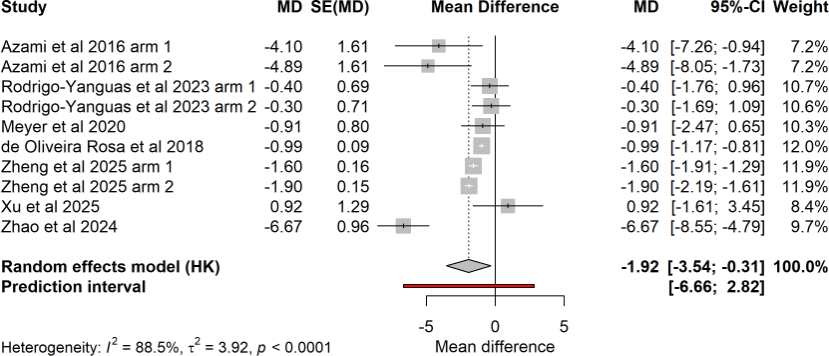
Forest plot of the SNAP-IV-PHI (Swanson, Nolan, and Pelham Rating Scale [Version IV] hyperactivity-impulsivity) subscale score [10,38,40,45,55,56,63]. MD: mean difference.

**Figure 8.**
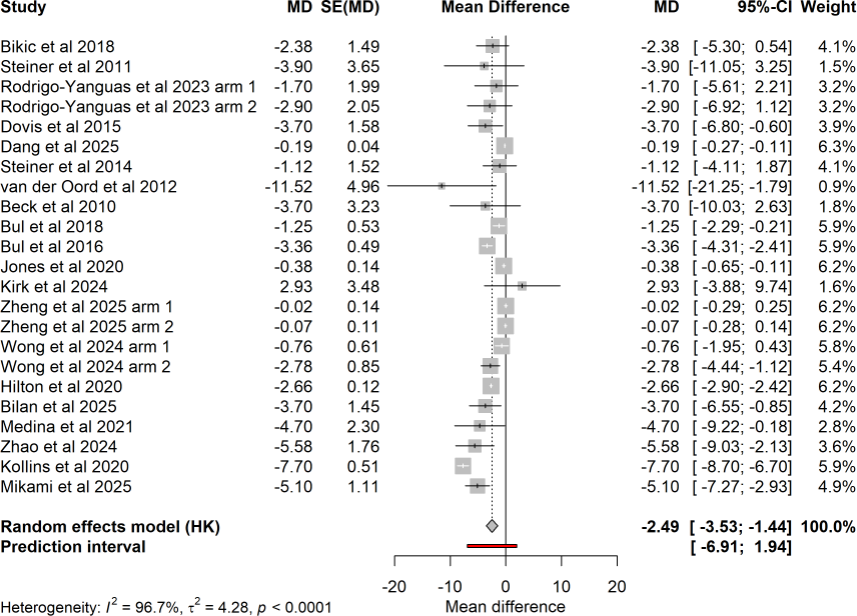
Forest plot of the BRIEF (Behavior Rating Inventory of Executive Function) scale score [10-12,39-42,45-47,49-54,57-59,62]. MD: mean difference.

Collectively, these findings substantiate that, based on CI estimates, DTx confer a clear overall average advantage over control conditions in improving core symptoms and executive functions in children and adolescents with ADHD. However, when interpreted in conjunction with the PI results, this advantage was robustly generalizable only for the ADHD-RS, whereas the PI for both the SNAP-IV (including both PI and PHI subscales) subscales and the BRIEF crossed the null value. This discrepancy underscores the need for clinicians to exercise caution when extrapolating these aggregate findings to individual patients in real-world practice. Detailed results are presented in Figures 5–8.

### Results of NMA

#### Efficacy of Different DTx Modalities on the Core Symptoms of ADHD in Children and Adolescents

This study elucidated the effects of different DTx modalities on core symptoms in children and adolescents with ADHD by analyzing data from the ADHD-RS and SNAP-IV (including both PI and PHI subscales).

For the ADHD-RS outcome measure, the included studies comprised 6 studies using single-task DTx, 1 study using cognitive-motor dual-task DTx, 1 study using AI-integrated single-task DTx, and 5 studies using AI-integrated cognitive-motor dual-task DTx, involving a total of 1642 patients.

**Figure 9.**
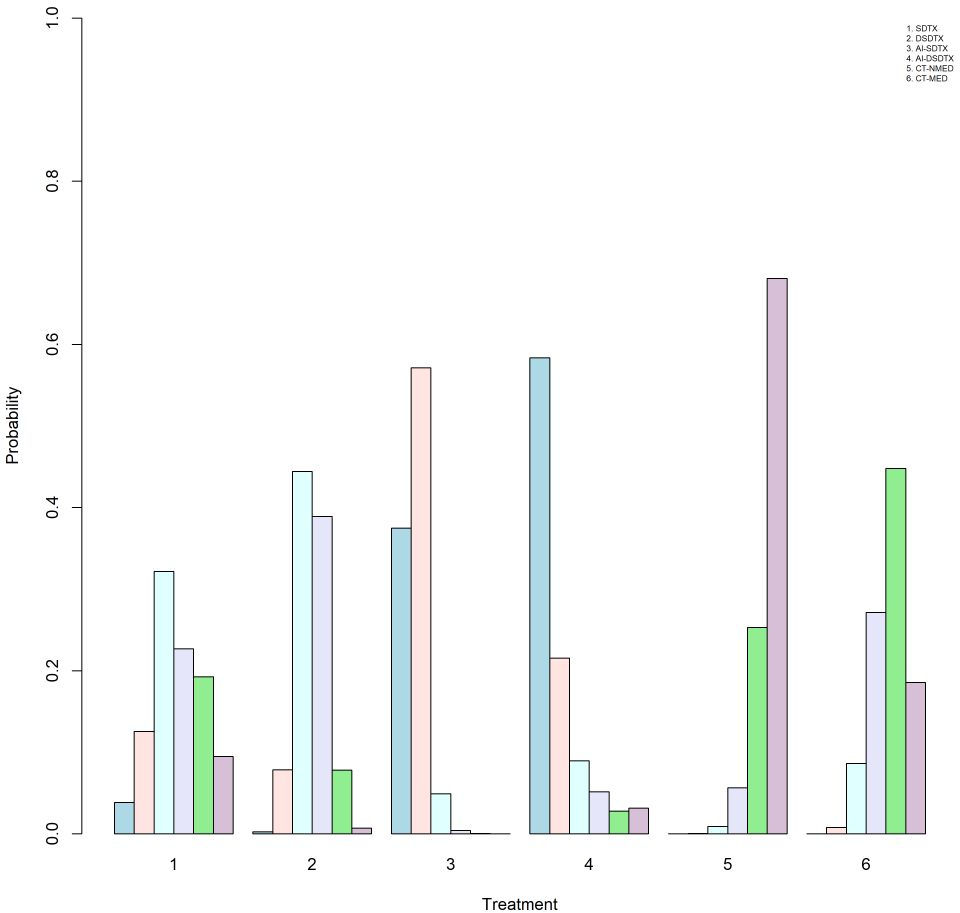
SUCRA chart of ADHD-RS scores. ADHD-RS: Attention-Deficit/Hyperactivity Disorder Rating Scale; AI-DSDTx: AI-integrated cognitive-motor dual-task digital therapeutics; AI-SDTx: AI-integrated single-task digital therapeutics; CT-MED: conventional treatments (MED); CT-NMED: conventional treatments (non-MED); DSDTx: cognitive-motor dual-task digital therapeutics; SDTx: single-task digital therapeutics; SUCRA: surface under the cumulative ranking curve.

Based on the SUCRA values and ranking results, the probability of each intervention being optimal for improving ADHD-RS scores in children and adolescents with ADHD was ranked as follows: AI-integrated cognitive-motor dual-task DTx (SUCRA 57.5%)>AI-integrated single-task DTx (SUCRA 22.5%)>single-task DTx (SUCRA 16.5%)>cognitive-motor dual-task DTx (SUCRA 2.1%)>conventional treatments (non-MED) (SUCRA 1.4%)>conventional treatments (MED) (SUCRA 0%). These findings suggest that AI-integrated cognitive-motor dual-task DTx had the highest probability of being the optimal intervention for improving inattention symptoms as measured by the ADHD-RS. However, the direct pairwise comparisons between the top-ranked AI-integrated cognitive-motor dual-task DTx (according to SUCRA) and the other DTx modalities did not reach statistical significance, suggesting that this ranking should be interpreted with caution (see Figure 9).

League table results (Table 3) demonstrated that, in terms of improving ADHD-RS scores in children and adolescents with ADHD, AI-integrated cognitive-motor dual-task DTx exhibited statistically significant advantages over both conventional therapies: compared to conventional treatments (non-MED), the effect size (MD) was −4.67 (95% CrI −6.50 to −2.85); compared to conventional treatments (MED), the effect size (MD) was −3.03 (95% CrI −5.59 to −0.47). Additionally, single-task DTx also showed significant improvement compared to conventional treatments (non-MED) (MD −2.69, 95% CrI −4.95 to −0.43).

**Table 3.** League table of treatment effects on ADHD-RS^a^ scores.^b^

	CT-NMED^c^	CT-MED^d^	DSDTx^e^	SDTx^f^	Al-DSDTx^g^	Al-SDTx^h^
CT-NMED	—^j^	—	—	—	—	—
CT-MED	−1.65 (−4.49 to 1.20)	—	—	—	—	—
DSDTx	−2.20 (−5.60 to 1.20)	−0.55 (−4.99 to 3.88)	—	—	—	—
SDTx	−2.69 (−4.95 to −0.43)	−1.05 (−3.41 to 1.32)	−0.49 (−4.57 to 3.59)	—	—	—
Al-DSDTx	−4.67 (−6.50 to −2.85)	−3.03 (−5.59 to −0.47)	−2.47 (−6.33 to 1.38)	−1.98 (−4.16 to 0.20)	—	—
Al-SDTx	−6.64 (−13.47 to 0.20)	−4.99 (−11.21 to 1.23)	−4.44 (−12.07 to 3.20)	−3.94 (−10.59 to 2.71)	−1.96 (−8.68 to 4.76)	—

a ADHD-RS: Attention-Deficit/Hyperactivity Disorder Rating Scale.

b Values are expressed as mean difference and 95% credible intervals.

c CT-NMED: conventional treatments (non-MED).

d CT-MED: conventional treatments (MED).

e DSDTx: cognitive-motor dual-task digital therapeutics.

f SDTx: single-task digital therapeutics.

g AI-DSDTx: AI-integrated cognitive-motor dual-task digital therapeutics.

h AI-SDTx: AI-integrated single-task digital therapeutics.

i Not applicable.

For studies included in this analysis that used the SNAP-IV as an outcome measure, particular focus was given to the PI and PHI subscales. A total of 5 studies using single-task DTx, 1 study using cognitive-motor dual-task DTx, 1 study using AI-integrated single-task DTx, and 1 study using AI-integrated cognitive-motor dual-task DTx were included, involving 468 patients.

**Figure 10.**
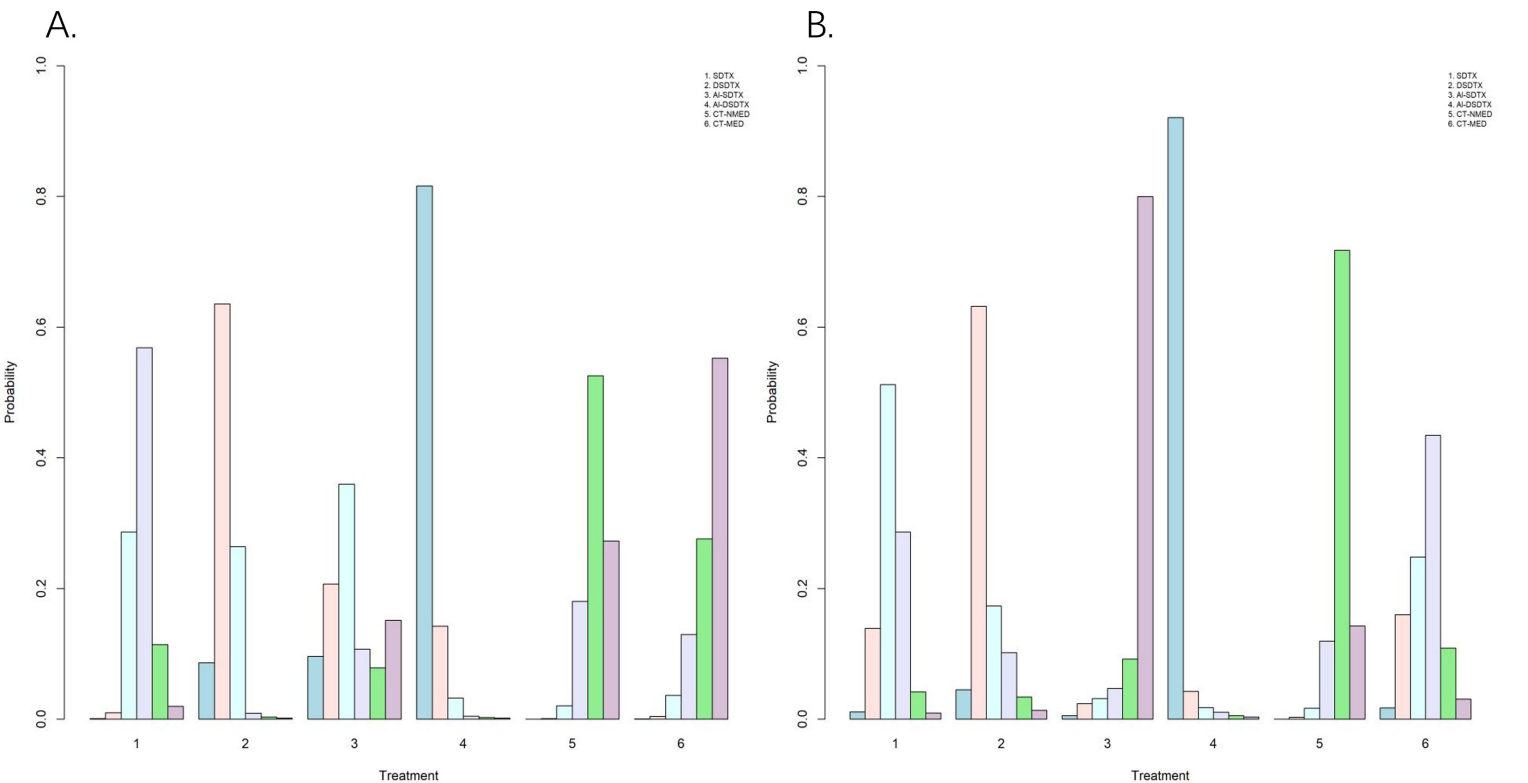
SUCRA (surface under the cumulative ranking curve) charts of (A) SNAP-IV-PI subscale scores and (B) SNAP-IV-PHI subscale scores. AI-DSDTx: AI-integrated cognitive-motor dual-task digital therapeutics; AI-SDTx: AI-integrated single-task digital therapeutics; CT-MED: conventional treatments (MED); CT-NMED: conventional treatments (non-MED), DSDTx: cognitive-motor dual-task digital therapeutics; SDTx: single-task digital therapeutics; SNAP-IV-PHI: Swanson, Nolan, and Pelham Rating Scale (Version IV) hyperactivity-impulsivity subscale; SNAP-IV-PI: Swanson, Nolan, and Pelham Rating Scale (Version IV) inattention subscale.

Based on the SUCRA values and ranking results, the probability of each intervention being optimal for improving scores on the SNAP-IV-PI subscale in children and adolescents with ADHD was ranked as follows: AI-integrated cognitive-motor dual-task DTx (SUCRA 82.5%)>AI-integrated single-task DTx (SUCRA 9.2%)>cognitive-motor dual-task DTx (SUCRA 8.1%)>single-task DTx (SUCRA 0.08%)>conventional treatments (MED) (SUCRA 0.03%)>conventional treatments (non-MED) (SUCRA 0%). These findings suggest that AI-integrated cognitive-motor dual-task DTx had the highest probability of being the optimal intervention for improving inattention symptoms as measured by the SNAP-IV-PI (see Figure 10A).

League table results (Table 4) showed that AI-integrated cognitive-motor dual-task DTx had statistically significant advantages over both conventional nonpharmacological therapy (MD –5.27, 95% CrI –7.89 to –2.65) and conventional pharmacotherapy (MD –5.58, 95% CrI –8.76 to –2.39) in improving SNAP-IV-PI scores. Cognitive-motor dual-task DTx also showed significant improvements relative to conventional treatments (non-MED) (MD –3.25, 95% CrI –4.86 to –1.64) and conventional treatments (MED) (MD –3.55, 95% CrI –5.78 to –1.33). However, single-task DTx was significantly less effective than AI-integrated cognitive-motor dual-task DTx (MD –4.41, 95% CrI –7.31 to –1.52) and cognitive-motor dual-task DTx (MD –2.39, 95% CrI –4.00 to –0.78). However, the direct pairwise comparisons between this modality and both AI-integrated single-task DTx (MD –3.36, 95% CrI –8.52 to 1.80) and cognitive-motor dual-task DTx (MD –2.02, 95% CrI –5.09 to 1.05) did not reach statistical significance, and therefore this ranking should be interpreted with caution.

Regarding improvement in SNAP-IV-PHI subscale scores, the SUCRA ranking results were as follows: AI-integrated cognitive-motor dual-task DTx (SUCRA 92.6%)>cognitive-motor dual-task DTx (SUCRA 4.3%)>single-task DTx (SUCRA 2.2%)>AI-integrated single-task DTx (SUCRA 0.8%)>conventional treatments (MED) (SUCRA 0.1%)>conventional treatments (non-MED) (SUCRA 0%). These findings indicate that AI-integrated cognitive-motor dual-task DTx had the highest probability of being the optimal intervention for improving hyperactivity-impulsivity symptoms as measured by the SNAP-IV-PHI (see Figure 10).

League table results (Table 5) demonstrated that, in terms of improving SNAP-IV-PHI scores, AI-integrated cognitive-motor dual-task DTx exhibited statistically significant advantages compared to conventional treatments (non-MED) (MD –6.67, 95% CrI –9.65 to –3.69), conventional treatments (MED) (MD –6.84, 95% CrI –10.37 to –3.31), single-task DTx (MD –5.88, 95% CrI –9.15 to –2.61), AI-integrated single-task DTx (MD –8.59, 95% CrI –13.13 to –4.05), and cognitive-motor dual-task DTx (MD –4.57, 95% CrI –8.05 to –1.10). Cognitive-motor dual-task DTx also showed significant improvements compared to conventional treatments (non-MED) (MD –2.10, 95% CrI –3.88 to –0.31) and AI-integrated single-task DTx (MD –4.02, 95% CrI –7.88 to –0.15).

**Table 4.** League table of treatment effects on SNAP-IV-PI^a^ subscale scores.^b^

	CT-MED^c^	CT-NMED^d^	SDTx^e^	Al-SDTx^f^	DSDTx^g^	Al-DSDTx^h^
CT-MED	—^i^	—	—	—	—	—
CT-NMED	–0.31 (–2.12 to 1.50)	—	—	—	—	—
SDTx	–1.16 (–2.88 to 0.55)	–0.86 (–2.10 to 0.39)	—	—	—	—
Al-SDTx	–2.22 (–7.01 to 2.58)	–1.91 (–6.35 to 2.53)	–1.05 (–5.67 to 3.56)	—	—	—
DSDTx	–3.55 (–5.78 to –1.33)	–3.25 (–4.86 to –1.64)	–2.39 (–4.00 to –0.78)	–1.34 (–6.07 to 3.39)	—	—
Al-DSDTx	–5.58 (–8.76 to –2.39)	–5.27 (–7.89 to –2.65)	–4.41 (–7.31 to –1.52)	–3.36 (–8.52 to 1.80)	–2.02 (–5.09 to 1.05)	—

a SNAP-IV-PI: Swanson, Nolan, and Pelham Rating Scale (Version IV) inattention subscale.

b Values are presented as mean difference and 95% credible intervals.

c CT-MED: conventional treatments (MED).

d CT-NMED: conventional treatments (non-MED).

e SDTx: single-task DTx.

f AI-SDTx: AI-integrated single-task DTx.

g DSDTx: cognitive-motor dual-task DTx.

h AI-DSDTx: AI-integrated cognitive-motor dual-task DTx.

i Not applicable.

**Table 5.** League table of treatment effects on SNAP-IV-PHI^a^ subscale scores.^b^

	Al-SDTx^c^	CT-MED^d^	CT-NMED^e^	SDTx^f^	DSDTx^g^	Al-DSDTx^h^
Al-SDTx	—^i^	—	—	—	—	—
CT-MED	–1.75 (–5.66 to 2.17)	—	—	—	—	—
CT-NMED	–1.92 (–5.35 to 1.51)	–0.17 (–2.06 to 1.72)	—	—	—	—
SDTx	–2.71 (–6.39 to 0.97)	–0.96 (–2.76 to 0.83)	–0.79 (–2.13 to 0.56)	—	—	—
DSDTx	–4.02 (–7.88 to –0.15)	–2.27 (–4.65 to 0.11)	–2.10 (–3.88 to –0.31)	–1.31 (–3.09 to 0.48)	—	—
Al-DSDTx	–8.59 (–13.13 to –4.05)	–6.84 (–10.37 to –3.31)	–6.67 (–9.65 to –3.69)	–5.88 (–9.15 to –2.61)	–4.57 (–8.05 to –1.10)	—

a SNAP-IV-PHI: Swanson, Nolan, and Pelham Rating Scale (Version IV) hyperactivity-impulsivity subscale.

b Values are presented as mean difference and 95% credible intervals.

c AI-SDTx: AI-integrated single-task DTx.

d CT-MED: conventional treatments (MED).

e CT-NMED: conventional treatments (non-MED).

f SDTx: single-task DTx.

g DSDTx: cognitive-motor dual-task DTx.

h AI-DSDTx: AI-integrated cognitive-motor dual-task DTx.

i Not applicable.

#### Efficacy of Different DTx Modalities on Executive Functions in Children and Adolescents With ADHD

This study elucidated the effects of different DTx modalities on executive functions in children and adolescents with ADHD by analyzing data from the BRIEF.

**Figure 11.**
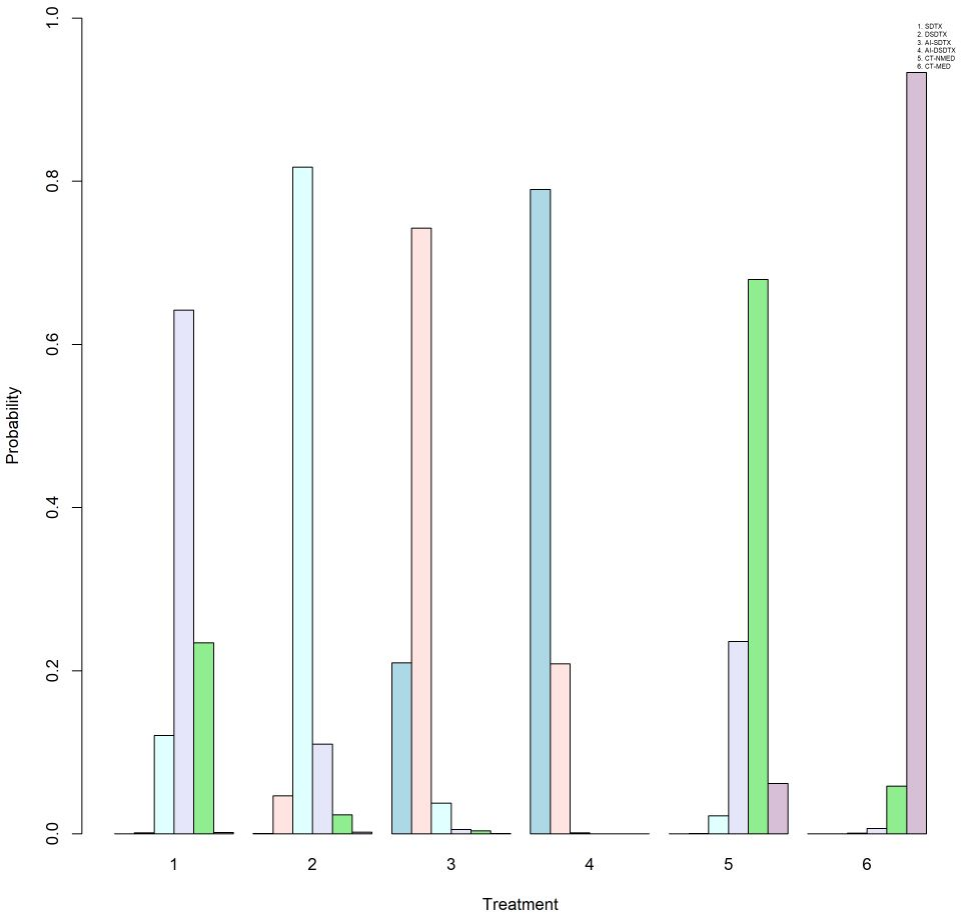
SUCRA (surface under the cumulative ranking curve) chart of BRIEF scores. AI-DSDTx: AI-integrated cognitive-motor dual-task DTx; AI-SDTx: AI-integrated single-task DTx; BRIEF: Behavior Rating Inventory of Executive Function; CT-MED: Conventional treatments (MED); CT-NMED: Conventional treatments (non-MED); DSDTx: cognitive-motor dual-task DTx; SDTx: single-task DTx.

For studies included in this analysis that used the BRIEF as an outcome measure, a total of 15 studies using single-task DTx, 3 studies using cognitive-motor dual-task DTx, 2 studies using AI-integrated single-task DTx, and 3 studies using AI-integrated cognitive-motor dual-task DTx were included, involving 1927 patients. Based on the SUCRA values and ranking results, the probability of each intervention being optimal for improving BRIEF scores in children and adolescents with ADHD was ranked as follows: AI-integrated cognitive-motor dual-task DTx (SUCRA 84.4%)>AI-integrated single-task DTx (SUCRA 14.8%)>cognitive-motor dual-task DTx (SUCRA 0.1%)>single-task DTx (SUCRA 0.002%)>conventional treatments (non-MED) (SUCRA 0.001%)>conventional treatments (MED) (SUCRA 0%). These findings indicate that AI-integrated cognitive-motor dual-task DTx had the highest probability of being the optimal intervention for improving executive functions as measured by the BRIEF (see Figure 11). However, the direct pairwise comparison between this modality and the third-ranked AI-integrated single-task DTx did not reach statistical significance (MD −2.04, 95% CrI −5.98 to 1.89); therefore, this ranking should be interpreted with caution (see Table 6).

This study evaluated the comparative effects of various interventions on executive functions (assessed by the BRIEF) in children and adolescents with ADHD through direct comparisons within the NMA (league table; Table 6). The specific results were as follows: in terms of improving BRIEF scores, AI-integrated cognitive-motor dual-task DTx demonstrated the most extensive significant advantages compared to conventional treatments (non-MED) (MD −6.88, 95% CrI −9.29 to −4.47), conventional treatments (MED) (MD −7.75, 95% CrI −10.06 to −5.43), single-task DTx (MD −6.11, 95% CrI −8.30 to −3.92), and cognitive-motor dual-task DTx (MD −5.35, 95% CrI −7.95 to −2.75). Furthermore, AI-integrated single-task DTx also significantly outperformed conventional treatments (non-MED) (MD −4.84, 95% CI −8.41 to −1.26), conventional treatments (MED) (MD −5.70, 95% CrI −9.26 to −2.15), and single-task DTx (MD −4.07, 95% CrI −7.34 to −0.80). Additionally, cognitive-motor dual-task DTx demonstrated significant efficacy compared to both conventional therapies: conventional treatments (non-MED) (MD −1.53, 95% CrI −3.04 to −0.01) and conventional treatments (MED) (MD −2.39, 95% CrI −4.46 to −0.33). Similarly, single-task DTx showed a significant advantage over conventional treatments (MED) (MD −1.64, 95% CrI: −3.04 to −0.24).

Of note, the AI-integrated single-task DTx node was supported by only 4 studies (n=69), substantially fewer than the other modality nodes. Although retained as a distinct category within our classification framework, the corresponding NMA estimates and SUCRA rankings should be interpreted with caution.

**Table 6.** League table of treatment effects on BRIEF^a^ scores.^b^

	CT-MED^c^	CT-NMED^d^	SDTx^e^	DSDTx^f^	Al-SDTx^g^	Al-DSDTx^h^
CT-MED	—^i^	—	—	—	—	—
CT-NMED	−0.87 (−2.76 to 1.03)	—	—	—	—	—
SDTx	−1.64 (−3.04 to −0.24)	−0.77 (−2.21 to 0.67)	—	—	—	—
DSDTx	−2.39 (−4.46 to −0.33)	−1.53 (−3.04 to −0.01)	−0.76 (−2.36 to 0.85)	—	—	—
Al-SDTx	−5.70 (−9.26 to −2.15)	−4.84 (−8.41 to −1.26)	−4.07 (−7.34 to −0.80)	−3.31 (−6.95 to 0.33)	—	—
Al-DSDTx	−7.75 (−10.06 to −5.43)	−6.88 (−9.29 to −4.47)	−6.11 (−8.30 to −3.92)	−5.35 (−7.95 to −2.75)	−2.04 (−5.98 to 1.89)	—

a BRIEF: Behavior Rating Inventory of Executive Function.

b Values are presented as mean difference and 95% credible intervals.

c CT-MED: conventional treatments (MED).

d CT-NMED: conventional treatments (non-MED).

e SDTx: single-task DTx.

f DSDTx: cognitive-motor dual-taskDTx.

g AI-SDTx: AI-integrated single-task DTx.

h AI-DSDTx: AI-integrated cognitive-motor dual-task DTx.

i Not applicable.

### Heterogeneity Assessment

For the SNAP-IV (both PI and PHI subscales) and the BRIEF, although the pooled mean effects were statistically significant (with 95% CI excluding the null), the corresponding 95% PI all crossed zero. This indicates that the observed treatment effects for these outcomes may exhibit considerable heterogeneity when generalized to future pediatric populations with different characteristics. In contrast, the 95% PI for the ADHD-RS did not cross zero, confirming that the findings for this outcome are characterized by robust external validity and low between-study heterogeneity (Figure 5).

Descriptively, *I*^2^ values were below 50% for the ADHD-RS and exceeded 50% for the BRIEF, SNAP-IV-PI, and SNAP-IV-PHI. These values are presented for descriptive purposes only and were not used to classify the severity of heterogeneity (see Figures S7A-S7D in Multimedia Appendix 3). Subgroup analyses, sensitivity analyses, and meta-regression were subsequently performed to explore potential sources of between-study variation.

### Subgroup Analysis and Meta-Regression

To explore potential sources of heterogeneity, meta-regression analyses were conducted, with year of publication, intervention duration, and mean participant age as covariates. The results showed that none of these 3 variables significantly moderated the pooled effect sizes for the BRIEF or the SNAP-IV (both PI and PHI subscales; see Figures S8A-S10C in Multimedia Appendix 3).

Given that heterogeneity tests revealed significant heterogeneity across outcome measures on the SNAP-IV (both PI and PHI subscales) and the BRIEF, further subgroup analyses were conducted to explore potential sources of heterogeneity in these outcomes. Subgroup analyses were performed based on intervention duration, mean participant age, and sex ratio.

Subgroup analyses stratified by intervention duration revealed substantially reduced heterogeneity within specific subgroups. Specifically, for the SNAP-IV-PI subscale, no significant heterogeneity was observed in the 7-week subgroup (*I*^2^=0%; MD −1.11, 95% CI −1.23 to −0.98). However, aside from the 7-week subgroup, heterogeneity remained substantial (*I*^2^>50%) in other intervention duration subgroups (eg, 4-week and 5-week), suggesting that the explanatory role of intervention duration for heterogeneity may be confined to specific duration intervals. For the SNAP-IV-PHI subscale, the 3-month subgroup also demonstrated low heterogeneity (*I*^2^=0%; MD −0.97, 95% CI −1.30 to −0.64). For the BRIEF, heterogeneity was substantially reduced in the 6-week subgroup (*I*^2^=0%; MD −5.23, 95% CI −9.46 to −1.00). Moreover, the overall results of these subgroup analyses were statistically significant (all *P*<.001; Figures S11A-S11C in Multimedia Appendix 3). These findings suggest that intervention duration represents a potential source of heterogeneity, influencing scores on the SNAP-IV (both PI and PHI subscales) and the BRIEF (see Figures S11A-S11C in Multimedia Appendix 3).

Subgroup analyses based on mean participant age categorized participants into 3 subgroups: preschool children (4 years≤age<6 years), school-aged children (6 years≤age<12 years), and adolescents (12 years≤age≤17 years). The results demonstrated substantially reduced heterogeneity within specific subgroups. Specifically, for the SNAP-IV-PI subscale, no significant heterogeneity was observed in the adolescent subgroup (*I*^2^=0%; MD −0.83, 95% CI −1.11 to −0.54). For the BRIEF, heterogeneity was substantially reduced in the adolescent subgroup (*I*^2^=0%; MD −2.50, 95% CI −4.88 to −0.11). Moreover, the overall results of these subgroup analyses were statistically significant (*P*<.001; Figures S12A-S12C in Multimedia Appendix 3). These findings suggest that mean participant age represents a potential source of heterogeneity influencing scores on the SNAP-IV-PI subscale and the BRIEF. Although heterogeneity was substantially reduced in the adolescent subgroup for the BRIEF (*I*^2^=0%), heterogeneity remained substantial in the preschool and school-aged children subgroups, indicating that the moderating effect of age on executive function outcomes may be primarily concentrated in adolescent populations. For the SNAP-IV-PHI subscale, heterogeneity was not substantially reduced in any subgroup after stratification by age (*I*^2^>50% in all subgroups), suggesting that age is not a primary source of heterogeneity for this outcome dimension (see Figures S12A-S12C in Multimedia Appendix 3).

Given the higher prevalence of ADHD in male children and adolescents, studies in which the proportion of female participants reached 30% were defined in this study as the group with a notable proportion of female participants. Accordingly, in the subgroup analyses based on sex ratio, studies were categorized into a male group (proportion of male participants >70%) and a female group (proportion of female participants ≥30%). After stratification by sex ratio, the results of subgroup analyses for all outcome measures indicated that sex ratio did not emerge as a potential source of heterogeneity (see Figures S13A-S13C in Multimedia Appendix 3).

### Sensitivity Analysis

To evaluate the robustness of the pooled results, sensitivity analyses were conducted using the leave-one-out method. The results revealed that for the SNAP-IV-PI subscale, the pooled effect size changed substantially after excluding the study by Zheng et al [50], suggesting that this study exerted a dominant influence on the outcome for this measure. Similarly, for the SNAP-IV-PHI subscale, the stability of the pooled results was affected following the exclusion of the study by de Oliveira Rosa et al [61]. For the BRIEF, the exclusion of the study by Hilton et al [64] also had a substantial impact on the overall effect size. These findings indicate that these particular studies represent potential sources of the high heterogeneity observed in the corresponding outcome measures. This influence may be attributable to the relatively small sample sizes of these studies (all with group sample sizes <30), which could lead to disproportionate weighting in the pooled analyses and consequently contribute to increased heterogeneity (see Figures S14A-S14C in Multimedia Appendix 3).

To assess whether the SUCRA-based ranking of AI-integrated cognitive-motor dual-task DTx as the top modality was robust to the inclusion of studies with a high risk of bias, we conducted a sensitivity analysis excluding the high-risk-of-bias studies, the results of which are presented in Multimedia Appendix 3 (Figures S15A-S15D). In this analysis, AI-integrated cognitive-motor dual-task DTx remained the highest-ranked intervention across all outcomes, consistent with the primary analysis, indicating that the SUCRA-based ranking of this modality as the optimal intervention is robust to the exclusion of high-risk-of-bias studies.

### Small-Study Effects

**Figure 12.**
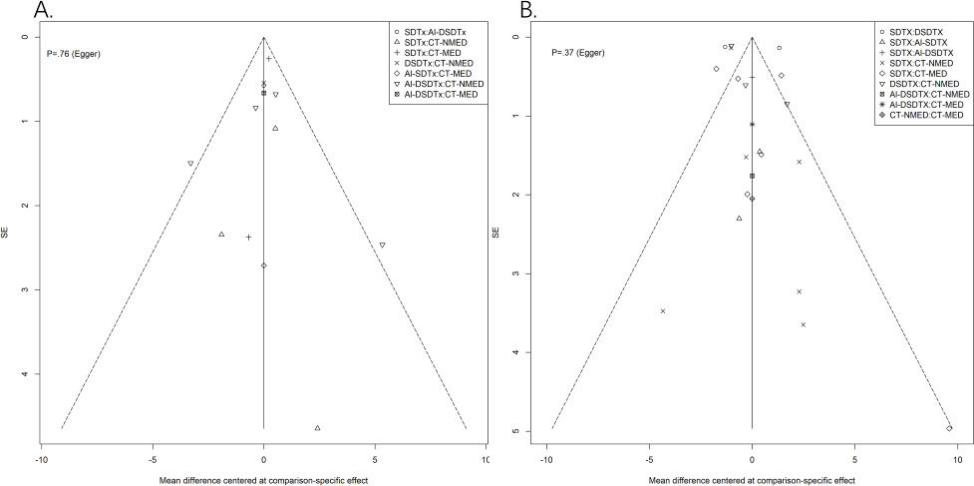
Egger test plot of (A) the ADHD-RS and (B) the BRIEF. ADHD-RS: Attention-Deficit/Hyperactivity Disorder Rating Scale; AI-DSDTx: AI-integrated cognitive-motor dual-task digital therapeutics; AI-SDTx: AI-integrated single-task digital therapeutics; BRIEF: Behavior Rating Inventory of Executive Function; CT-MED: conventional treatments (MED); CT-NMED: conventional treatments (non-MED); DSDTx: cognitive-motor dual-task digital therapeutics; SDTx: single-task digital therapeutics.

Funnel plots and Egger test were used to evaluate small-study effects. Assessments were performed for outcome measures with more than 10 included studies, specifically the ADHD-RS and BRIEF. No obvious asymmetry was observed in the funnel plots for either outcome. Egger test revealed no significant evidence of small-study effects for studies using the ADHD-RS (*P*=.76) or the BRIEF (*P*=.38) as outcome measures (all *P*>.05; see Figure 12).

### Certainty of Evidence

For the ADHD-RS and SNAP-IV (both the PI and PHI subscales), the certainty of evidence comparing various DTx modalities with conventional interventions was rated as low. In contrast, for the BRIEF, the certainty of evidence was rated as moderate. These GRADE ratings indicate that the findings of this study should be interpreted with caution (see Table 1).

## Discussion

This study is explicitly an efficacy-focused systematic review and NMA and represents the first NMA conducted within the dual framework of task design and AI empowerment to systematically compare the relative therapeutic efficacy of 4 DTx modalities for ADHD in pediatric populations. Moreover, this study overcomes the limitations of pairwise comparisons inherent in traditional meta-analyses and provides evidence that may inform clinical practice.

### Comparative Efficacy of Different DTx Modalities on Core Symptoms

SUCRA rankings consistently placed AI-integrated cognitive-motor dual-task DTx first across the ADHD-RS and both SNAP-IV subscales, with statistically significant advantages over both nonpharmacological and pharmacological conventional treatments. For the ADHD-RS, the effect relative to nonpharmacological therapy exceeded the established minimal important difference (MID) threshold of 4 points [75]. The advantage over medication did not reach this threshold; moreover, this comparison relies on indirect evidence and should be interpreted cautiously [75]. Notably, the modality showed a particularly pronounced benefit on hyperactivity-impulsivity symptoms, with a substantially greater magnitude of improvement than other DTx modalities—a domain where conventional nonpharmacological interventions typically produce only small to moderate effects [36].

The superior performance of this combined modality likely reflects synergies between cognitive-motor dual-task design and AI-driven personalization [11,76]. From the perspective of task design, this dual-task paradigm possesses a unique neurophysiological theoretical foundation compared to traditional single-task training. According to Klingberg et al [11], the dual-task paradigm requires the brain to coordinate motor output (eg, balance control and fine motor skills) while simultaneously executing cognitive tasks (eg, attentional control and working memory updating) [11]. Although speculative, this high-intensity cognitive load may more effectively activate frontostriatal circuitry and influence dopaminergic neurotransmission, thereby potentially enhancing neuroplasticity [11,76]. However, the present study did not directly measure neural activity or neurotransmitter function, and this mechanistic interpretation remains speculative. Future neuroimaging studies (eg, functional magnetic resonance imaging) are needed to test these hypothesized mechanisms.

From the perspective of AI empowerment, AI-integrated DTx leverage machine learning algorithms to analyze patients’ task performance indicators in real time (eg, reaction time, accuracy rates, and attentional fluctuation patterns) and dynamically adjust task difficulty and training content accordingly [77]. This may help patients remain within an optimal training zone where their capabilities are appropriately challenged, potentially maximizing training benefits [8,14]. The study by Bilan et al [14] demonstrated that AI-driven adaptive algorithms significantly enhance treatment adherence and the magnitude of cognitive improvement in children with ADHD. All DTx modalities also incorporate game-based elements (eg, points, levels, rewards, and narrative contexts) as supportive components. Although game elements are not considered direct therapeutic agents, they play a critical role in enhancing user engagement, motivation, and treatment adherence, particularly in pediatric populations [78]. The present study extends this understanding by suggesting that, during dual-task execution, AI algorithms may identify cognitive bottlenecks (eg, periods of attentional lapses) and dynamically adjust task parameters to target these vulnerable areas, thereby potentially facilitating more precise modulation of neural circuits [13]. However, this hypothesized mechanism remains to be empirically verified, and future neuroimaging studies are needed to test these hypotheses.

### Comparative Efficacy of Different DTx Modalities on Executive Functions

For executive functions measured by the BRIEF, AI-integrated cognitive-motor dual-task DTx also achieved the highest SUCRA ranking and significantly outperformed both conventional nonpharmacological and pharmacological controls.

Executive function is a multidimensional construct encompassing inhibitory control, working memory updating, and cognitive flexibility [79]. Single-task DTx typically target isolated cognitive domains, limiting their capacity for broad transfer to real-world executive function [80]. In contrast, cognitive-motor dual-task training inherently requires coordinated deployment of multiple executive subcomponents, mirroring the cognitive demands of daily functioning [81]. This capacity for multitask coordination is itself a critical manifestation of executive functions [82]. According to the executive function development theory of Diamond [83], training with higher ecological validity produces stronger and more generalizable transfer effects. Cognitive-motor dual-task training parallels real-life situations such as “walking while thinking,” and this dual-task paradigm may produce broader transfer effects by promoting the integrated training of executive function subcomponents [84]. One potential mechanism underlying this broader transfer is that dual-task training may enhance the connectivity between prefrontal regions and motor-related cortical areas, thereby facilitating the integration of cognitive and motor processes [85]. However, this mechanistic interpretation remains speculative in the absence of direct neurophysiological evidence from the present study.

The integration of AI further amplifies this advantage. The AI-integrated cognitive-motor dual-task DTx modalities included in this study (eg, AKL-T01, BrainFit, and SDT-001) all use AI-driven adaptive algorithms capable of dynamically adjusting cognitive task difficulty and motor task complexity based on patients’ real-time performance [10,12,67]. Beyond the general adaptive adjustment described earlier, a possible mechanism by which AI integration contributes to executive function enhancement is that adaptive algorithms may continuously calibrate the task cognitive workload to match individual fluctuating attention and working memory capacity, thereby preventing cognitive overload or under-engagement [13]. Nevertheless, this hypothesis awaits direct testing in future studies using neuroimaging or electrophysiological measures.

### Interpretation of Findings

When interpreting the findings of this NMA, 3 methodological factors should be considered simultaneously: heterogeneity, risk of bias, and certainty of evidence.

PI-based heterogeneity assessments revealed differential generalizability across outcome measures. The PI for the ADHD-RS were consistently narrow and excluded zero, indicating that the improvements in core ADHD symptoms are robust and reproducible. In contrast, the PI for both the SNAP-IV (PI and PHI subscales) and the BRIEF all crossed zero, suggesting that improvements in inattention, hyperactivity-impulsivity, and executive functions may vary considerably across populations, thus exhibiting limited generalizability to individual patients. Subgroup and sensitivity analyses further identified intervention duration, age, and several small-sample studies as potential sources of heterogeneity; however, the intervention rankings based on SUCRA values remained consistent. Collectively, these findings indicate that AI-integrated cognitive-motor dual-task DTx represents a statistically significant average benefit (a central tendency) rather than a universally fixed therapeutic effect.Therefore, when tailoring individual clinical care, practitioners should consider patient-specific factors such as age and treatment duration.

Regarding risk of bias, 40.6% of the included RCTs were rated as having a high risk of bias, primarily due to inadequate reporting of randomization methods or the inability to blind parents as outcome assessors. A sensitivity analysis excluding the high-risk-of-bias studies confirmed that AI-integrated cognitive-motor dual-task DTx remained the highest-ranked intervention across all outcomes, consistent with the primary analysis, indicating that the SUCRA-based ranking of this modality as the optimal intervention is robust to bias exclusion.

Regarding GRADE certainty, the evidence for ADHD-RS and SNAP-IV outcomes was rated as low certainty, whereas the evidence for the BRIEF was rated as moderate certainty. The low ratings were primarily driven by serious imprecision (wide CI crossing the null for some comparisons) and a high risk of bias in some included studies. These GRADE ratings indicate that the findings should be interpreted with caution.

In summary, while the SUCRA-based rankings consistently favored AI-integrated cognitive-motor dual-task DTx across all outcomes, the strength of this evidence is constrained by low to moderate GRADE certainty, substantial heterogeneity in some outcomes, and a notable proportion of studies with a high risk of bias. The absolute effect estimates should therefore be interpreted with the above limitations in mind.

### Clinical Implications

These findings have several important clinical implications and should be considered within a human-centered digital health framework[86]. When conditions permit, AI-integrated cognitive-motor dual-task DTx may be considered as a nonpharmacological intervention option for children and adolescents with ADHD, particularly for those with prominent hyperactivity-impulsivity symptoms or those who experience adverse effects from pharmacotherapy. Notably, however, this conclusion is based on probabilistic SUCRA rankings, and the differences between this modality and other DTx modalities did not reach statistical significance across several outcome dimensions. Therefore, clinical decisions should integrate individual patient factors, including age, symptom profile, comorbidities, family preferences, and intervention accessibility[86].

DTx should be viewed as supportive rather than substitutive components of comprehensive ADHD care[86]. They are most effective when integrated into a multimodal treatment plan that may include pharmacotherapy, behavioral therapy, and educational support[86]. Successful implementation requires careful consideration of usability, engagement, and family acceptability [87].

Clinical oversight and governance are equally critical for the safe and effective use of DTx in pediatric populations [88]. Health care providers should monitor treatment progress, adherence, and potential adverse effects and adjust the treatment plan as needed [88]. Clear protocols for responsibility assignment, outcome documentation, and family communication should be established when integrating DTx into routine clinical care pathways.

Moreover, the SUCRA advantage of AI-integrated cognitive-motor dual-task DTx must be interpreted with attention to within-category heterogeneity, as this aggregated modality spans multiple technological implementations with distinct mechanisms of action [10,12,13,52,66,68-71]. Without head-to-head comparisons across subtypes, whether this ranking reflects a general property or is subtype driven remains unclear. Beyond outcome-level effect sizes, evaluating the AI layer itself, including adaptive difficulty adjustment, real-time performance analytics, and personalized feedback, requires component-specific methodological frameworks [89]. Similarly, translating screen-based pediatric interventions into routine clinical care calls for regulatory approaches that address not only clinical efficacy but also data privacy, algorithmic transparency, age-appropriate design, and commercial incentives [90]. These considerations should inform future implementation strategies as AI-enabled DTx move from controlled trials to real-world practice.

### Limitations

Several limitations should be acknowledged. First, 40.6% of included studies had a high risk of bias, mainly due to inadequate reporting of randomization and blinding procedures, although sensitivity analyses suggested limited impact on overall rankings. Second, within-modality heterogeneity in AI algorithms, delivery platforms, and training protocols may reduce the precision of pooled effect estimates. Third, the relatively small number of AI-integrated DTx studies limits statistical power and introduces potential publication bias risk. Fourth, reliance on parent-reported outcomes introduces potential expectancy bias and may not fully capture school-based or objective cognitive performance. Fifth, subgroup analyses by ADHD subtype could not be performed due to insufficient reporting of outcomes stratified by diagnostic subtype in the included studies [91]. Sixth, small-study effects could not be formally assessed for SNAP-IV subscales due to the limited number of studies. Seventh, insufficient data on adherence and long-term follow-up prevented the evaluation of dose-response relationships and durability of effects. Eighth, trial conditions may not fully reflect real-world implementation factors such as family support, device access, and connectivity. Additionally, some degree of misclassification cannot be excluded given the variation in AI algorithms and delivery formats across studies, which may add uncertainty to network estimates.

### Future Directions

Future large-scale head-to-head RCTs should compare AI-integrated DTx against pharmacotherapy and behavioral therapy using standardized outcomes. Mechanistic studies (eg, functional magnetic resonance imaging, magnetoencephalography, and electroencephalography) should validate the synergistic hypothesis. Prediction models are needed to identify patient characteristics (eg, age, sex, and ADHD subtype) predicting differential response. Pragmatic trials should assess real-world effectiveness, including family support, device access, and clinical oversight. Implementation research should examine integration strategies for DTx into pediatric ADHD care, including responsibility assignment and outcome documentation [88]. Human-centered design studies should assess usability and acceptability among children, adolescents, and families. Finally, extended follow-up (≥6 months) should determine the long-term maintenance of DTx effects on academic achievement and social functioning.

### Conclusions

This dual-dimension classification framework NMA advances the evidence base for ADHD DTx by systematically comparing 4 modalities and integrating ranking, heterogeneity quantification via PI, and GRADE certainty assessment. AI-integrated cognitive-motor dual-task DTx emerges as the highest-ranked intervention for both core symptoms and executive functions, with preliminary evidence of synergy between dual-task design and AI personalization and with intervention duration and age identified as potential sources of heterogeneity. Despite low to moderate evidence certainty and remaining methodological limitations, these findings provide actionable guidance for clinical practice and future DTx development, pending confirmation from well-designed head-to-head trials.

## Supplementary material

10.2196/95043 Multimedia Appendix 1 Search strategy.

10.2196/95043 Multimedia Appendix 2 Literature quality evaluation results.

10.2196/95043 Multimedia Appendix 3 Figures depicting the Bayesian inconsistency test charts, frequential inconsistency assessment, convergent diagnostic plots, convergence trace and density plots, heterogeneity test charts, meta-regression plots, subgroup analysis charts, subscale sensitivity analysis charts, sensitivity analysis for risk of bias stratification for various scales and subscales.

10.2196/95043 Checklist 1 PRISMA checklist.

## Abbreviations

ADHD: attention-deficit/hyperactivity disorder
ADHD-RS: Attention-Deficit/Hyperactivity Disorder Rating Scale
BRIEF: Behavior Rating Inventory of Executive Function
CrI: credible intervals
DIC: deviance information criterion
DL: DerSimonian-Laird
DSM-5: Diagnostic and Statistical Manual of Mental Disorders (Fifth Edition)
DTx: digital therapeutics
HKSJ: Hartung-Knapp-Sidik-Jonkman
ICD-10: International Classification of Diseases, Tenth Revision
MD: mean difference
MID: minimal important difference
NMA: network meta-analysis
PI: prediction intervals
PICOS: participants, intervention, comparator, outcome, and studies
PRISMA: Preferred Reporting Items for Systematic Reviews and Meta-Analyses
PRISMA-S: Preferred Reporting Items for Systematic Reviews and Meta-Analyses Literature Search Extension
PSRF: potential scale reduction factor
RCT: randomized controlled trial
REML: restricted maximum likelihood
SNAP-IV: Swanson, Nolan, and Pelham Rating Scale, Version IV
SNAP-IV-PHI: Swanson, Nolan, and Pelham Rating Scale (Version IV) hyperactivity-impulsivity subscale
SNAP-IV-PI: Swanson, Nolan, and Pelham Rating Scale (Version IV) inattention subscale
SUCRA: surface under the cumulative ranking curve
WISC-IV: Wechsler Intelligence Scale for Children, Fourth Edition
