# Supplementary material for: Multimodal Digital Therapeutics Enhanced by Task Design and AI for Attention-Deficit/Hyperactivity Disorder Core Symptoms and Executive Functions in Children and Adolescents: Systematic Review and Network Meta-Analysis of Randomized Controlled Trials
Source: J Med Internet Res. 2026 Aug 18;28:e95043. doi: 10.2196/95043 (PMC13487414; doi:10.2196/95043)
Supplement: Supplementary file 1 [file jmir-v28-e95043-s001.docx]

**Search strategy**

**PubMed/Medline search strategy**

#1 “Attention Deficit Disorder with Hyperactivity” [Mesh] OR (“ADHD” OR “Attention Deficit Hyperactivity Disorders” OR “Attention-deficit/Hyperactivity Disorder” OR “Hyperkinetic Syndrome”) [Title/Abstract]

#2 “Child” [Mesh] OR “Adolescent” [Mesh] OR (“Child*” OR “Adolescent*” OR “Pediatric*” OR “Paediatric*” OR “Young people”) [Title/Abstract]

#3 #1 OR #2

#4 “Video games” [Mesh] OR “Exergaming” [Mesh] OR “Digital health” [Mesh] OR (“Digital therapeutics” OR “Digital intervention” OR “Digital game” OR “Serious game” OR “Video game” OR “Computer game” OR “Gamification” OR “Exergame”) [Title/Abstract]

#5 (“Single task” OR “Dual task” OR “Double task”) [Title/Abstract]

#6 “Artificial intelligence” [Mesh] OR (“AI” OR “Artificial intelligence”) [Title/Abstract]

#7 #4 OR #5 OR #6

#8 “Executive function” [Mesh] OR “Cognition” [Mesh] OR (“Executive functions” OR “Executive control” OR “Cognitive function” OR “Cognitions”) [Title/Abstract]

#9 #3 AND #7 AND #8

**PsycINFO search strategy**

#1 (“Attention Deficit Disorder with Hyperactivity” OR “ADHD” OR “Attention Deficit Hyperactivity Disorder” OR “Attention Deficit Disorder” OR “hyperkinetic”).SU

#2 (“Childhood” OR “ Adolescence” OR “Child*” OR “Adolescent*” OR “Pediatric*” OR “Paediatric*” OR “Youth ”OR “Teen*” OR “Young people”).SU

#3 #1 OR #2

#4 (“Video Games” OR “Exergaming” OR “Digital health” OR “Digital therapeutics” OR “Digital intervention*” OR “Serious game*” OR “Video game*” OR Exergame* OR “Computer game*” OR “Game-based” OR “Gamification*”).SU

#5 (“Single task*” OR “Dual task*” OR “Double task*”).SU

#6 (“Artificial intelligence” OR “AI” OR “Machine learning”).SU

#7 #4 OR #5 OR #6

#8 (“Cognition” OR “Executive function” OR “Executive function*” OR “Cognition function” OR “Cognitions” OR “Executive control”).SU

#9 #3 AND #7 AND #8

**Web of Science search strategy**

#1 TS = (“Attention Deficit Disorder with Hyperactivity” OR “ADHD” OR “Attention Deficit Hyperactivity Disorders” OR “Attention-deficit/Hyperactivity Disorder”)

#2 TS = (“Child” OR “Adolescent” OR “Pediatric” OR “Paediatric” OR “Young people”)

#3 #1 OR #2

#4 TS = (“Digital therapeutics” OR “Digital intervention” OR “Video games” OR “Exergaming” OR “Digital Health” OR “Digital game” OR “Serious game” OR “Video game” OR “Computer game” OR “Gamification” OR “Exergame”)
#5 TS = (“Single task” OR “Dual task” OR “Double task”)

#6 TS = (“Artificial intelligence” OR “AI”)

#7 #4 OR #5 OR #6

#8 TS = (“Executive function” OR “Executive functions” OR “Executive control” OR “Cognitive function” OR “Cognitions”)

#9 #3 AND #7 AND #8

**Cochrane Library search strategy**

#1 MeSH descriptor: [Attention Deficit Disorder with Hyperactivity] explode all trees
#2 MeSH descriptor: [Child] explode all trees

#3 (Pediatric):ti,ab,kw OR (Paediatric):ti,ab,kw

#4 MeSH descriptor: [Adolescent] explode all trees

#5 (Young people):ti,ab,kw

#6 #1 OR #2 OR #3 OR #4 OR #5

#7 MeSH descriptor: [Digital health] explode all trees

#8 (Digital therapeutics):ti,ab,kw OR (Digital intervention):ti,ab,kw OR (Digital game):ti,ab,kw
#9 MeSH descriptor: [Video Games] explode all trees

#10 (Serious game):ti,ab,kw OR (Computer game):ti,ab,kw
#11 MeSH descriptor: [Exergaming] explode all trees

#12 (Exergame):ti,ab,kw OR (Gamification):ti,ab,kw

#13 MeSH descriptor: [Artificial intelligence] explode all trees

#14 (Artificial intelligence):ti,ab,kw OR (AI):ti,ab,kw

#15 (Single task):ti,ab,kw OR (Dual task):ti,ab,kw OR (Double task):ti,ab,kw

#16 #7 OR #8 OR #9 OR #10 OR #11 OR #12 OR #13 OR #14
#17 (Executive function):ti,ab,kw OR (Executive functions):ti,ab,kw OR (Executive control):ti,ab,kw

#18 #6 AND #16 AND #17

**EMBASE search strategy**

#1 ' Attention Deficit Disorder with Hyperactivity '/exp OR ' ADHD ':ab,kw,ti OR ' Attention Deficit Hyperactivity Disorders ':ab,kw,ti OR ' Attention-deficit/Hyperactivity Disorder ':ab,kw,ti

#2 'Child'/exp OR 'Adolescent'/exp OR 'Pediatric':ab,kw,ti OR 'Paediatric':ab,kw,ti OR 'Young people':ab,kw,ti

#3 #1 OR #2

#4 'Digital health'/exp OR 'Digital therapeutics':ab,kw,ti OR 'Digital intervention':ab,kw,ti OR 'Digital game':ab,kw,ti OR 'Video games':ab,kw,ti OR 'Serious game':ab,kw,ti OR 'Computer game':ab,kw,ti OR 'Gamification':ab,kw,ti OR 'Exergame':ab,kw,ti

#5 'Artificial intelligence'/exp OR 'AI':ab,kw,ti

#6 'Single task'/exp OR 'Dual task':ab,kw,ti OR 'Double task':ab,kw,ti

#7 #4 OR #5 OR #6

#8 'Executive function'/exp OR 'Executive functions'/exp OR 'Executive control'/exp OR 'Cognitive function':ab,kw,ti OR 'Cognitions':ab,kw,ti

#9 #3 AND #7 AND #8

**Scopus** **search strategy**

#1 (“Attention Deficit Disorder with Hyperactivity” OR “ADHD” OR “Attention Deficit Hyperactivity Disorders” OR “Attention-deficit/Hyperactivity Disorder” OR “Hyperkinetic Syndrome”)

#2 (“Child” OR “Adolescent” OR “Pediatric” OR “Young people”)

#3 #1 OR #2

#4 (“Digital therapeutics” OR “Digital health” OR “Digital intervention” OR “Digital game” OR “Serious game” OR “Video game” OR “Computer game” OR “Gamification” OR “Exergame” OR “Exergaming”)

#5 (“Single task” OR “Dual task” OR “Double task”)

#6 (“Artificial intelligence” OR “AI”)

#7 #4 OR #5 OR #6

#8 (“Executive function” OR “Executive functions” OR “Executive control” OR “Cognitive function” OR “Cognitions”)

#9 #3 AND #7 AND #8

**ProQuest** **search strategy**

#1 mainsubject(“Attention Deficit Disorder with Hyperactivity” OR “ADHD” OR “Attention Deficit Hyperactivity Disorders” OR “Attention-deficit/Hyperactivity Disorder” OR “Hyperkinetic Syndrome” OR “Child” OR “Adolescent” OR “Pediatric” OR “Paediatric” OR “Young people”)

#2 mainsubject(“Digital therapeutics” OR “Digital health” OR “Digital intervention” OR “Digital game” OR “Serious game” OR “Video game” OR “Computer game” OR “Gamification” OR “Exergame” OR “Exergaming” OR “Single task” OR “Dual task” OR “Double task” OR “Artificial intelligence” OR “AI”)

#3 mainsubject(“Executive function” OR “Executive functions” OR “Executive control” OR “Cognitive function” OR “Cognitions”)

#4 #1 AND #2 AND #3

**Clinical Trials.gov search strategy**

Condition/disease: ADHD (Attention-Deficit/Hyperactivity Disorder)

Intervention/treatment: Digital therapy, Exergaming, Digital interactive technology, Exergame, Serious game, Computer game

Outcome measure: core symptoms and executive function

Location: –

Study Status: All studies

Sex: All

Age: Child (birth - 17)

Study Phase: –

Study Type: Interventional, Observational

Study Results: With results

Study Documents: –

Date Range：2000.01.01-2026.05.22
