# Supplementary material for: Multimodal Digital Therapeutics Enhanced by Task Design and AI for Attention-Deficit/Hyperactivity Disorder Core Symptoms and Executive Functions in Children and Adolescents: Systematic Review and Network Meta-Analysis of Randomized Controlled Trials
Source: J Med Internet Res. 2026 Aug 18;28:e95043. doi: 10.2196/95043 (PMC13487414; doi:10.2196/95043)
Supplement: Supplementary file 2 [file jmir-v28-e95043-s002.docx]

**TableS1.** Literature Quality Evaluation Table

| Study | Randomisation Process | Deviations from intended interventios | Mising outcome data | Measurement of the outcome | Selection of the report results | Overall Bias |
| --- | --- | --- | --- | --- | --- | --- |
| Bikic et al (2017) | Some conerns | Low | High | Low | High | High |
| Azami et al (2016) | Some conerns | High | Low | High | Some conerns | High |
| Bikic et al (2018) | Low | Some conerns | Low | Low | Low | Some conerns |
| Steiner et al (2011) | High | High | Some conerns | Some conerns | Low | High |
| Rodrigo-Yanguas et al (2023) | Low | Low | Low | Low | Low | Low |
| Dovis et al (2015) | Some conerns | High | Low | Low | Low | High |
| Dang et al (2025) | High | Some conerns | Low | Some conerns | Low | High |
| Steiner et al (2014) | Some conerns | Low | Low | Some conerns | Some conerns | Some conerns |
| van der Oord et al (2012) | Low | Low | Low | Low | Low | Low |
| Beck et al (2010) | High | Low | Low | Some conerns | Some conerns | High |
| Bul et al (2018) | Some conerns | High | Low | Some conerns | Low | High |
| Bul et al (2016) | Some conerns | Low | Low | Low | Low | Some conerns |
| Meyer et al (2020) | High | Low | Low | Low | Some conerns | High |
| de Oliveira Rosa et al (2018) | Some conerns | High | Low | Low | Low | High |
| Jones et al (2020) | Low | Low | Low | Low | Low | Low |
| Bioulac et al (2020) | High | Some conerns | Low | Low | Low | High |
| Kirk et al (2024) | Low | Low | Low | Low | Low | Low |
| Zheng et al (2025) | Low | Low | Low | Low | Low | Low |
| Wong et al (2024) | Low | Low | Low | Low | Low | Low |
| Hilton et al (2020) | Low | Low | Low | Low | Low | Low |
| Kim et al (2022) | Some conerns | Some conerns | Low | Some conerns | Some conerns | Some conerns |
| Bilan et al (2025) | Some conerns | Some conerns | Low | Low | Low | Some conerns |
| Medina et al (2021) | Low | Low | Low | Low | Low | Low |
| Xu et al (2025) | Some conerns | Some conerns | Low | Low | Low | Some conerns |
| Zhao et al (2024) | Some conerns | Some conerns | High | High | Some conerns | High |
| Kollins et al (2020) | Low | Some conerns | Low | Low | Low | Some conerns |
| Kollins et al (2021) | High | Some conerns | Low | Low | Low | High |
| Mikami et al (2025) | Low | Low | Low | Low | Low | Low |
| Lim et al (2019) | Low | Low | Low | Low | Low | Low |
| McDermot et al (2020) | Some conerns | High | Low | Low | Low | High |
| Qian et al (2018) | Low | Low | Low | Low | Low | Low |
| Mikami et al (2025) | Low | Low | Low | Low | Low | Low |
