# Supplementary material for: Multimodal Digital Therapeutics Enhanced by Task Design and AI for Attention-Deficit/Hyperactivity Disorder Core Symptoms and Executive Functions in Children and Adolescents: Systematic Review and Network Meta-Analysis of Randomized Controlled Trials
Source: J Med Internet Res. 2026 Aug 18;28:e95043. doi: 10.2196/95043 (PMC13487414; doi:10.2196/95043)
Supplement: Supplementary file 3 [file jmir-v28-e95043-s004.doc]

| **Section and Topic** | **Item #** | **Checklist item** | **Location**  **where item is reported** |
| --- | --- | --- | --- |
| **TITLE** | | |  |
| Title | 1 | Identify the report as a systematic review. | 1 |
| **ABSTRACT** | | |  |
| Abstract | 2 | See the PRISMA 2020 for Abstracts checklist. | 1-2 |
| **INTRODUCTION** | | |  |
| Rationale | 3 | Describe the rationale for the review in the context of existing knowledge. | 2-3 (Introduction) |
| Objectives | 4 | Provide an explicit statement of the objective(s) or question(s) the review addresses. | 3 (Introduction, last paragraph) |
| **METHODS** | | |  |
| Eligibility criteria | 5 | Specify the inclusion and exclusion criteria for the review and how studies were grouped for the syntheses. | 3-4 (Eligibility Criteria section) |
| Information sources | 6 | Specify all databases, registers, websites, organizations, reference lists and other sources searched or consulted to identify studies. Specify the date when each source was last searched or consulted. | 3 (Information Sources and Search Strategy section) |
| Search strategy | 7 | Present the full search strategies for all databases, registers and websites, including any filters and limits used. | 3 (Information Sources and Search Strategy section full strategy in Multimedia Appendix 1) |
| Selection process | 8 | Specify the methods used to decide whether a study met the inclusion criteria of the review, including how many reviewers screened each record and each report retrieved, whether they worked independently, and if applicable, details of automation tools used in the process. | 3 (Selection Process section) |
| Data collection process | 9 | Specify the methods used to collect data from reports, including how many reviewers collected data from each report, whether they worked  independently, any processes for obtaining or confirming data from study investigators, and if applicable, details of automation tools used in the process. | 4 (Data Collection Process section) |
| Data items | 10a | List and define all outcomes for which data were sought. Specify whether all results that were compatible with each outcome domain in each study were sought (e.g. for all measures, time points, analyses), and if not, the methods used to decide which results to collect. | 4 (Eligibility Criteria, Outcomes subsection) |
|  | 10b | List and define all other variables for which data were sought (e.g. participant and intervention characteristics, funding sources). Describe any assumptions made about any missing or unclear information. | 4 (Data Items section; study characteristics in Table 2) |
| Study risk of bias assessment | 11 | Specify the methods used to assess risk of bias in the included studies, including details of the tool(s) used, how many reviewers assessed each study and whether they worked independently, and if applicable, details of automation tools used in the process. | 4 (Risk of Bias Assessment in Studies section) |
| Effect measures | 12 | Specify for each outcome the effect measure(s) (e.g. risk ratio, mean difference) used in the synthesis or presentation of results. | 5 (Summary measures section) |
| Synthesis methods | 13a | Describe the processes used to decide which studies were eligible for each synthesis (e.g. tabulating the study intervention characteristics and comparing against the planned groups for each synthesis (item #5)). | 5-6 (Synthesis Methods section) |
|  | 13b | Describe any methods required to prepare the data for presentation or synthesis, such as handling of missing summary statistics, or data conversions. | 5-6 (Synthesis Methods section) |
|  | 13c | Describe any methods used to tabulate or visually display results of individual studies and syntheses. | 5-6 (Synthesis Methods section) |
|  | 13d | Describe any methods used to synthesize results and provide a rationale for the choice(s). If meta-analysis was performed, describe the model(s), method(s) to identify the presence and extent of statistical heterogeneity, and software package(s) used. | 5-6 (Synthesis Methods section) |
|  | 13e | Describe any methods used to explore possible causes of heterogeneity among study results (e.g. subgroup analysis, meta-regression). | 5-6 (Synthesis Methods section) |
|  | 13f | Describe any sensitivity analyses conducted to assess robustness of the synthesized results. | 5-6 (Synthesis Methods section) |
| Reporting bias assessment | 14 | Describe any methods used to assess risk of bias due to missing results in a synthesis (arising from reporting biases). | 6 (Reporting Bias Assessment section) |
| Certainty  assessment | 15 | Describe any methods used to assess certainty (or confidence) in the body of evidence for an outcome. | 6 (Certainty Assessment section) |

| **Section and Topic** | **Item #** | **Checklist item** | **Location**  **where item is reported** |
| --- | --- | --- | --- |
| **RESULTS** | | |  |
| Study selection | 16a | Describe the results of the search and selection process, from the number of records identified in the search to the number of studies included in the review, ideally using a flow diagram. | 8 (Study Selection section; flow diagram in Figure 1) |
|  | 16b | Cite studies that might appear to meet the inclusion criteria, but which were excluded, and explain why they were excluded. | 8 (Study Selection section; full exclusion list available upon request) |
| Study  characteristics | 17 | Cite each included study and present its characteristics. | 9-14 (Study Characteristics section; detailed in Table 2 |
| Risk of bias in studies | 18 | Present assessments of risk of bias for each included study. | 15-16 (Risk of Bias in Studies section; summary in Figures 2-3, detailed in Multimedia Appendix 3) |
| Results of  individual studies | 19 | For all outcomes, present, for each study: (a) summary statistics for each group (where appropriate) and (b) an effect estimate with its precision (e.g. confidence/credible interval), ideally using structured tables or plots. | 17-19 (Forest plots: Figures 5-8 and Multimedia Appendix 3 Figures S2a-S2d |
| Results of syntheses | 20a | For each synthesis, briefly summarize the characteristics and risk of bias among contributing studies. | 16-26 (Each outcome section includes summary of included studies) |
|  | 20b | Present results of all statistical syntheses conducted. If meta-analysis was done, present for each the summary estimate and its precision (e.g. confidence/credible interval) and measures of statistical heterogeneity. If comparing groups, describe the direction of the effect. | 19-24 (Network Meta-analysis Results section; figures in Figures 9-11, tables in Tables 3-6) |
|  | 20c | Present results of all investigations of possible causes of heterogeneity among study results. | 24 (Subgroup Analysis and Meta-regression section; detailed in Multimedia Appendix 3 Figures S8A-S13C) |
|  | 20d | Present results of all sensitivity analyses conducted to assess the robustness of the synthesized results. | 25 (Sensitivity Analysis section; detailed in Multimedia Appendix 3 Figures S14A-S15D) |
| Reporting biases | 21 | Present assessments of risk of bias due to missing results (arising from reporting biases) for each synthesis assessed. | 25 (Small-Study Effects section; detailed in Figures 12) |
| Certainty of evidence | 22 | Present assessments of certainty (or confidence) in the body of evidence for each outcome assessed. | 26 (Certainty of Evidence section; detailed in Table 1) |
| **DISCUSSION** | | |  |
| Discussion | 23a | Provide a general interpretation of the results in the context of other evidence. | 26-28 (Discussion section) |
|  | 23b | Discuss any limitations of the evidence included in the review. | 28 (Limitations section) |
|  | 23c | Discuss any limitations of the review processes used. | 28 (Limitations section) |
|  | 23d | Discuss implications of the results for practice, policy, and future research. | 28 (Clinical Implications and Future Directions sections) |
| **OTHER INFORMATION** | | |  |
| Registration and protocol | 24a | Provide registration information for the review, including register name and registration number, or state that the review was not registered. | 6 (Protocol and Registration section; PROSPERO CRD420261304236) |
|  | 24b | Indicate where the review protocol can be accessed, or state that a protocol was not prepared. | 6 (Protocol and Registration section; registered but no public access link provided) |
|  | 24c | Describe and explain any amendments to information provided at registration or in the protocol. | 6 (Deviations from The Registered Protocol section) |
| Support | 25 | Describe sources of financial or non-financial support for the review, and the role of the funders or sponsors in the review. | 28 (Funding section) |
| Competing interests | 26 | Declare any competing interests of review authors. | 28 (Conflicts of Interest section) |
| Availability of data, code and other materials | 27 | Report which of the following are publicly available and where they can be found: template data collection forms; data extracted from included studies; data used for all analyses; analytic code; any other materials used in the review. | Relevant data are available upon request from the corresponding author or first author. |

*From:* Page MJ, McKenzie JE, Bossuyt PM, Boutron I, Hoffmann TC, Mulrow CD, et al. The PRISMA 2020 statement: an updated guideline for reporting systematic reviews. BMJ 2021;372:n71. doi:

10.1136/bmj.n71

For more information, visit:<http://www.prisma-statement.org/>
