# Supplementary material for: Multimodal Digital Therapeutics Enhanced by Task Design and AI for Attention-Deficit/Hyperactivity Disorder Core Symptoms and Executive Functions in Children and Adolescents: Systematic Review and Network Meta-Analysis of Randomized Controlled Trials
Source: J Med Internet Res. 2026 Aug 18;28:e95043. doi: 10.2196/95043 (PMC13487414; doi:10.2196/95043)
Supplement: Supplementary file 4 [file jmir-v28-e95043-s003.docx]

|   **Figure S1A. Bayesian Inconsistency Test Chart of ADHD-RS Scale** |   **Figure S1B. Bayesian Inconsistency Test Chart of SNAP-IV-PI Subscale** |
| --- | --- |
|   **Figure S1C. Bayesian Inconsistency Test Chart of SNAP-IV-PHI Subscale** |   **Figure S1D. Bayesian Inconsistency Test Chart of BRIEF Scale** |

Abbreviations: SDTx: Single-task digital therapy, DSDTx: Cognitive-motor dual-task digital therapy, AISDTx: AI-integrated single-task digital therapy, AIDSDTx: AI-integrated cognitive-motor dual-task digital therapy, CTNMED: Conventional treatments (non-MED), CTMED: Conventional treatments (MED)

|   **Figure S2A. Frequentist Inconsistency Assessment for the ADHD-RS subscale** |   **Figure S2B. Frequentist Inconsistency Assessment for the SNAP-IV-PI subscale** |
| --- | --- |
|   **Figure S2C.** **Frequentist Inconsistency Assessment for the SNAP-IV-PHI subscale** |   **Figure S2D. Frequentist Inconsistency Assessment for the BRIEF scale** |

Abbreviations: 1: Single-task digital therapy, 2: Cognitive-motor dual-task digital therapy, 3: AI-integrated single-task digital therapy, 4: AI-integrated cognitive-motor dual-task digital therapy, 5: Conventional treatments (non-MED), 6: Conventional treatments (MED)

|   **Figure S3A. Convergent Diagnostic Chart of the ADHD-RS Scale** |   **Figure S3B. Convergence Trace and Density Plot of the ADHD-RS Scale** |
| --- | --- |
| **Figure S4A.** **Convergence Diagnostic Plot of the SNAP-IV-PI Subscale** |   **Figure S4B. Convergence Traces and Density Plots of the SNAP-IV-PI Subscale** |
| **Figure S5A. Convergence Diagnostic Plot of the SNAP-IV-PHI Subscale** |   **Figure S5B. Convergence Trace and Density Plot of the SNAP-IV-PHI Subscale** |
| **Figure S6A. Convergence Diagnostic Plot of the BRIEF Scale** |   **Figure S6B. Convergence Trace and Density Plot of the BRIEF Scale** |

Abbreviations: 1: Single-task digital therapy, 2: Cognitive-motor dual-task digital therapy, 3: AI-integrated single-task digital therapy, 4: AI-integrated cognitive-motor dual-task digital therapy, 5: Conventional treatments (non-MED), 6: Conventional treatments (MED)

| **Figure S7A. Heterogeneity Test Chart of ADHD-RS Scale** |   **Figure S7B. Heterogeneity Test Chart of SNAP-IV-PI Subscale** |
| --- | --- |
|   **Figure S7C.** **Heterogeneity Test Chart of SNAP-IV-PHI Subscale** |   **Figure S7D. Heterogeneity Test Chart of the BRIEF Scale** |

Abbreviations: 1: Single-task digital therapy, 2: Cognitive-motor dual-task digital therapy, 3: AI-integrated single-task digital therapy, 4: AI-integrated cognitive-motor dual-task digital therapy, 5: Conventional treatments (non-MED), 6: Conventional treatments (MED)

|   **Figure S8A.** **Meta-regression graph of the publication years of the SNAP-IV-PI subscale** |   **Figure S8B. Meta-regression plot of intervention duration for the SNAP-IV-PI subscale** |   **Figure S8C. Meta-regression plot of the average age of subjects in the SNAP-IV-PI subscale** |
| --- | --- | --- |
|  |  |  |
|   **Figure S9A. Meta-regression graph of the publication years of the SNAP-IV-PHI subscale** |   **Figure S9B. Meta-regression plot of intervention duration for the SNAP-IV-PHI subscale** |   **Figure S9C. Meta-regression plot of the average age of subjects in the SNAP-IV-PHI subscale** |
|   **Figure S10A. Meta-regression plot of the publication year of the BRIEF scale** |   **Figure S10B. Meta-regression plot of intervention duration for the BRIEF scale** |   **Figure S10C. Meta-regression plot of the average age of subjects in the BRIEF scale** |

Abbreviations: 1: Single-task digital therapy, 2: Cognitive-motor dual-task digital therapy, 3: AI-integrated single-task digital therapy, 4: AI-integrated cognitive-motor dual-task digital therapy, 5: Conventional treatments (non-MED), 6: Conventional treatments (MED)

|   **Figure S11A. Subgroup Analysis Chart of Intervention Time for SNAP-IV-PI Subscale** |   **Figure S11B. Subgroup Analysis Chart of Intervention Time for SNAP-IV-PHI Subscale** | |   **Figure S11C.** **Subgroup Analysis Chart of Intervention Time for BRIEF Scale** | |  |
| --- | --- | --- | --- | --- | --- |
|   **Figure S12A.** **Subgroup Analysis Chart of Average Age of The Subjects for SNAP-IV-PI Subscale** | |   **Figure S12B. Subgroup Analysis Chart of Average Age of The Subjects for SNAP-IV-PHI Subscale** | |   **Figure S12C. Subgroup Analysis Chart of Average Age of The Subjects for BRIEF Scale** | |

Abbreviations: A: Preschool children group (4 years old ≤ age < 6 years old), B: School-age children group (6 years old ≤ age < 12 years old), C: Adolescent group (12 years old ≤ age ≤ 17 years old)

|   **Figure S13A. Subgroup Analysis Chart of Gender Ratio of Subjects for SNAP-IV-PI Subscale** |   **Figure S13B. Subgroup Analysis Chart of Gender Ratio of Subjects for SNAP-IV-PHI Subscale** |   **Figure S13C. Subgroup Analysis Chart of Gender Ratio of Subjects for BRIEF Scale** |
| --- | --- | --- |

|   **Figure S14A.** **SNAP-IV-PI Subscale Sensitivity Analysis Chart** |   **Figure S14B. Sensitivity Analysis Chart of the SNAP-IV-PHI Subscale** | |   **Figure S14C. Sensitivity Analysis Graph of the BRIEF Scale** |
| --- | --- | --- | --- |
|   **Figure S15A. Results of sensitivity analysis for risk of bias stratification of the ADHD-RS scale** | | **Figure S15B. Results of sensitivity analysis for risk of bias stratification of the SNAP-IV-PI scale** | |
| **Figure S15C. Results of sensitivity analysis for risk of bias stratification of the SNAP-IV-PHI scale** | | **Figure S15D. Results of sensitivity analysis for risk of bias stratification of the BRIEF scale** | |

Abbreviations: SDTx: Single-task digital therapy, DSDTx: Cognitive-motor dual-task digital therapy, AI-SDTx: AI-integrated single-task digital therapy, AI-DSDTx: AI-integrated cognitive-motor dual-task digital therapy, CT-NMED: Conventional treatments (non-MED), CT-MED: Conventional treatments (MED)
